# Targeting of Repeated Sequences Unique to a Gene Results in Significant Increases in Antisense Oligonucleotide Potency

**DOI:** 10.1371/journal.pone.0110615

**Published:** 2014-10-15

**Authors:** Timothy A. Vickers, Susan M. Freier, Huynh-Hoa Bui, Andrew Watt, Stanley T. Crooke

**Affiliations:** Department of Core Antisense Research, ISIS Pharmaceuticals, Inc., Carlsbad, California, United States of America; NIH, United States of America

## Abstract

A new strategy for identifying potent RNase H-dependent antisense oligonucleotides (ASOs) is presented. Our analysis of the human transcriptome revealed that a significant proportion of genes contain unique repeated sequences of 16 or more nucleotides in length. Activities of ASOs targeting these repeated sites in several representative genes were compared to those of ASOs targeting unique single sites in the same transcript. Antisense activity at repeated sites was also evaluated in a highly controlled minigene system. Targeting both native and minigene repeat sites resulted in significant increases in potency as compared to targeting of non-repeated sites. The increased potency at these sites is a result of increased frequency of ASO/RNA interactions which, in turn, increases the probability of a productive interaction between the ASO/RNA heteroduplex and human RNase H1 in the cell. These results suggest a new, highly efficient strategy for rapid identification of highly potent ASOs.

## Introduction

Antisense oligonucleotide (ASO) mediated reduction of targeted RNAs has been broadly exploited as both a research tool and in development of human therapeutics [1]. A better understanding of the molecular mechanisms by which ASOs reduce levels of targeted RNA is essential to the development of more potent and specific antisense therapeutics. The best understood mechanism through which short synthetic oligonucleotides modulate gene expression in mammalian cells is RNase H-dependent degradation of the targeted RNA [2], [3]. Two types of RNase H are expressed in human cells: RNase H1 and RNase H2. Experiments in which levels of these enzymes have been increased or reduced clearly demonstrate that the potency of ASOs positively correlates with the level and activity of RNase H1 but not H2 [4]. Specifically, increasing the levels of human RNase H1 in cells increases the potency of ASOs, whereas decreasing the levels of the enzyme leads to decreased ASO potency. RNase H1 is ubiquitously expressed in prokaryotes and eukaryotes and is found in the nucleus, cytoplasm, and mitochondria of eukaryotic cells [5]–[8]. Consistent with the cellular distribution of RNase H1, DNA-like ASOs effectively target the exonic and intronic regions of mRNAs as well as nuclear-retained RNAs [9], [10]. RNase H1 binds to the RNA-DNA heteroduplex through a hybrid binding domain located on the N terminus of the protein, with cleavage of the RNA occurring 7 to 10 nucleotides from the 5′-end of the RNA (approximately one helical turn) and requires a minimum of five consecutive DNA nucleotides hybridized to the RNA [4]. Modified second generation “gapmer” ASOs include an 8–14 base deoxynucleotide “gap”, flanked on either end with 2-modified nucleotides. The gap region promotes degradation of the target mRNA by RNase H-mediated cleavage while the flanking nucleotides enhance affinity for cognate RNA [11]. With a gapmer ASO, RNAse H1 cleavage typically occurs in the center of the gap, but cleavage sites and rates are influenced by sequence [12].

Despite the fact that ASOs of the same chemical class and length have very similar properties, ASOs targeting the same gene vary widely in potency [13]. There are several partial explanations for these substantial differences in potency. We have shown that mammalian RNase H1 is present at very low concentrations in mammalian cells [14] and that human RNase H1 has site and sequence preferences that influence potency. RNA structure also plays a significant role [15] as does target location within the pre-mRNA. ASO activity in introns is typically less robust than in exons and this activity is highly influenced by splicing rate [16], [17]. We have also shown that, on average, ASOs targeting nuclear-retained RNAs are more potent than those targeting cytoplasmic RNAs [18]. Recently, we have reported that phosphorothioate containing ASOs bind to a number of cellular proteins and these interactions alter subcellular localization and, in some cases, such as localization in PS bodies or paraspeckles, can affect potency [19], [20]. Additionally, at some sites, proteins that bind the ASO/RNA duplex can compete and bind to the site preferentially with RNase H1, limiting levels of activity [17].

In this manuscript, we consider another potential factor that might contribute to ASO activity and specificity: the presence of multiple cognate or nearly perfect sites in the target RNA. Receptor theory applies to ASOs hybridizing to RNAs just as it does to small molecules interacting with proteins, but there are subtle differences. For example, with most small molecules, scanning or sampling the environment to find the receptor site is extremely rapid. In contrast, scanning of nucleic acid space by ASOs is slower and correlates with affinity. Another potentially important difference is the possibility that RNA might contain multiple quasi-binding sites for an ASO. Since human RNase H1 is rate-limiting with respect to ASO activity in cells [14], in the cellular environment hybridization can only lead to cleavage when the ASO/RNA duplex is recognized and bound by RNase H. Increasing the frequency of ASO/RNA interactions on a particular target would therefore be expected to increase the likelihood of a productive cleavage reaction, ultimately resulting in increased potency of the ASO.

In the current study, we demonstrate that a significant fraction of the transcriptome contains repeated sequences present in only one target RNA. Our data clearly demonstrate that ASOs complementary to these repeat sequences are more potent than ASOs targeting single sites in the same mRNA. These repeats appear more frequently in introns, but occur throughout transcripts. Further, the mechanism underlying the increase in potency is simply that repeated target sites increase the probability of a productive interaction between ASO/RNA and human RNase H1. These results suggest that targeting ASOs to repeat sites should reduce the time and cost necessary to identify potent ASOs and, ultimately, will result in increased specificity of ASO-based therapeutics.

## Materials and Methods

### Identification and analysis of 16-mer repeat sequences in the human transcriptome

Human primary and processed transcript sequences from the Reference GRCh37.p13 Primary Assembly were obtained from Entrez. A multi-threaded and paralleled processing Java program was written to extract unique 16-mer sequences from the primary and processed transcripts. The locations of each 16-mer on primary and processed transcripts were then determined using Bowtie, an ultrafast and memory-efficient program for aligning short DNA sequences to the human genome [21]. 16-mer repeat sequences were identified as those that are mapped perfectly to two or more locations on a single transcript and matched only one gene. The coding DNA sequence (CDS) and 5′ and 3′ untranslated regions (UTR) of processed transcripts were defined based on current annotations in GenBank records. Distribution analyses of 16-mer repeat sequences were done using the R statistical software (http://www.r-project.org/).

### Preparation of antisense oligonucleotides

Synthesis and purification of phosphorothioate oligonucleotides was performed using an Applied Biosystems 380B automated DNA synthesizer as described previously [22]. All ASOs were “gapmers” 16–20 nucleotides in length with 2′-O-methoxyethyl (MOE) or constrained ethyl (cEt) [23] substitutions at the positions indicated in Supporting Information (Tables S3–S7).

### ASO treatment of cells and qRT/PCR analyses

Electroporation of ASOs into HepG2 cells was carried out using the HT-200 BTX electroporator with the ElectroSquare Porator (ECM 830) voltage source in 96-well electroporation plates (BTX, 2 mm; Harvard Apparatus). Cells were harvested 16 hours after electroporation. Cells were electroporated in the presence of ASOs at the indicated concentrations and plated. For lipid transfection of HeLa, T-REx-293, and HepG2 cells, cells were seeded in 96 well plates at ∼50% confluency then treated the following day with the indicated concentrations of ASO in Opti-MEM media (Invitrogen) containing 5 µg/ml Lipofectamine 2000 (Invitrogen) for 4 hours, as described previously [24]. Following transfection, cells were washed once with PBS, then fed with fresh growth media, and incubation was continued overnight. For minigene cell lines, 1 µg/ml tetracycline was added to the growth media to induce minigene transcription. For U4 and U6 snRNA reduction, SOD/GCGR cell lines were seeded in 6-cm dishes at 60% confluency then transfected with U4 ASO 479333 or U6 ASO 479338 [25] at 50 nM in Opti-MEM media containing 5 µg/ml Lipofectamine 2000. Cells were then trypsinized and seeded in 96 well plates. Cells were treated the following day with ASO at the indicated concentrations for 4 hours then fed with fresh growth media plus 1 µg/ml tetracycline for 4 hours to induce minigene expression.

Following ASO treatment, total RNA was purified using an RNeasy 3000 BioRobot (Qiagen). Target mRNA levels were assessed by qRT/PCR performed with 10 µl (∼10 ng) of total RNA and Express One-Step SuperScript qRT-PCR SuperMix reagents (Life Technologies) on an ABI StepOne Real Time PCR System (Applied Biosystems). The reverse transcription step was performed for 30 minutes at 48°C followed with 40 thermal cycles of 30 s at 94°C and 1 minute at 60°C. To avoid artifacts based upon well-to-well variation in cell number, mRNA levels were normalized to the total amount of RNA present in each reaction as determined by Ribogreen assay (Invitrogen) [26]. The sequences of the primers and probes are listed in Table S1.

Dose-response curves were generated by nonlinear regression analysis using GraphPad PRISM software. Best-fit values for the logIC_50_ of the dose response curves were analyzed by one-way ANOVA and compared using Bonferroni’s or or Newman-Keuls multiple comparison test. IC_50_ values for multiple repeat vs. single site screens were compared by Mann-Whitney U Test.

### Construction of SOD1/GCGR hybrid minigenes with multiple repeat sites

The preparation of the SOD-1 minigene construct has been previously described [14]. The GCGR 449884 site was inserted into the minigene at various positions. Nhe I restriction sites are found in exons 4 and 5 of the minigene construct. We used site-directed mutagenesis (SDM) to selectively mutate the first base of each of these sites from a G to a C using a QuikChange Lightning Site-Directed Mutagenesis Kit according to the manufacturer’s protocol. A third Nhe I site was also added to the intron by SDM changing the sequence GAGAGC to GCTAGC (mutated bases underlined). Synthetic DNA oligonucleotides, CTAG GGATCC GAGCTC GGTACC TGGGCACCTCGGGAACC and CTAG GGTTCCCGAGGTGCCCA GGTACC GAGCTC GGATCC, were annealed to create a double stranded adapter containing Kpn I, Sac I, and Bam H1 restriction sites, followed by a single GCGR site and flanked by Nhe I sticky ends, which was then ligated into the minigene at each Nhe I site to give pSOD/E4GR1X (exon 4), pSOD/E5GR1X (exon 5), or pSOD/I4GR1X (intron 4). To add multiple repeat sites, these plasmids were digested with Kpn I and NheI, then dsDNA oligonucleotides with complimentary ends were ligated directionally to give plasmids for expression of transcripts containing two tandem repeats (pSOD/E4GR2X, pSOD/E5GR2X, or pSOD/I4GR2X) and four tandem repeats (pSOD/E4GR4X, pSOD/E5GR4X, or pSOD/I4GR4X). Non-tandem repeats were inserted by SDM QuikChange Lightning Site-Directed Mutagenesis Kit. Primers were designed using the QuikChange Primer Design Program (Agilent Technologies, Inc.) such that the sequence TGGGCACCTCGGGAACC GGT (GCGR 449884 site plus Age I restriction site) would be inserted at positions 19, 334, 472, and 523 relative to the first SOD1 base of the minigene. Sequences of primers are given in Table S2. All minigene constructs were sequenced to confirm orientation, location, and number of repeats.

T-REx-293 cells were purchased from Invitrogen and cultured in DMEM supplemented with 10% fetal calf serum, streptomycin (0.1 µg/ml), penicillin (100 units/ml), and blasticidin (5 µg/ml). SOD/GCGR minigene plasmids were transfected into T-REx-293 cells using Effectene transfection reagent according to the manufacturer’s protocol (Qiagen). Cells containing the stably integrated minigene were selected in DMEM media containing 250 µg/ml Zeocin. Zeocin-resistant colonies were expanded and then tested for tetracycline-inducible expression by qRT/PCR using minigene specific primers and probes [14]. Cell lines stably expressing *E. coli* RNase H were generated as described previously [14].

### *In vitro* RNase H cleavage assays

The SOD/GCGR minigene is preceded by T7 RNA polymerase promoter [14]. Primers were designed to the vector sequence in pcDNA4 just upstream of the SOD/GCGR insert (GCTGTTTTGACCTCCATAGAA) and downstream of the GCGR repeat site in exon 4 (GAATGATGCAATGGTCTCCTG). pSOD/E4GR1X and pSOD/E4GR4X were used as templates to produce PCR fragments corresponding to the *GCGR* repeat sequence. T7 transcribed mRNA was generated from these DNA templates using a MEGAscript Kit according to the manufacturer’s protocol (Life Technologies, Cat# AM1334M). Following a 5-hour incubation at 37°C, 5 U of DNase I (Life Technologies) was added for 30 minutes at 37°C to remove template DNA. After the DNase treatment, the reaction was adjusted to 300 mM sodium acetate and extracted once with phenol/chloroform and once with chloroform. The RNA was then precipitated with 2 volumes of 100% ethanol. The RNA was 5′-end labeled with ^32^P by first dephosphorylating the transcript using 50 µg of RNA and 10 µL of Antarctic Phosphatase (New England Biolabs) in 100 µL 1X Antarctic Phosphatase buffer and incubating at 37°C for 60 minutes, followed by heat inactivation at 65°C for 5 minutes. The dephosphorylated RNA was purified using an RNeasy Mini Kit as per manufacturer’s instructions (Qiagen, Cat# 74104). The RNAs were 5′-end labeled using 40 pmol of RNA, 20 U of T4 polynucleotide kinase (Promega, Cat# M4101), 120 pmol [γ-32P] ATP (6000 ci/mmol) (Perkin Elmer, Cat# NEG035C005MC), 70 mM Tris, pH 7.6, 10 mM MgCl_2_ and 50 mM dithiothreitol. The kinase reaction was incubated at 37°C for 30 minutes and quenched using Gel Loading Buffer II (Life Technologies, Cat# AM8546G). The labeled transcript and oligoribonucleotides were purified by gel electrophoresis on a 12% denaturing polyacrylamide gel.

For cleavage assays the heteroduplex substrate was prepared as previously described [27]. Human RNase H1 (gift from H.Wu) was added to 100 nM substrate in a total volume of 100 µl of cleavage buffer (20 mM Tris-HCl, 50 mM NaCl, 2 mM MgCl_2_, and 0.1 mM TCEP, pH 7.5); 10 µl aliquots of the cleavage reaction were removed at time points ranging from 1.5 to 120 minutes and quenched by adding 5 µl of stop solution (8 M urea and 500 mM EDTA). The aliquots were heated at 90°C for 2 minutes, resolved on a 12% denaturing polyacrylamide gel, and the substrate and product bands were quantitated on an Amersham Biosciences PhosphorImager.

## Results

### Enhanced activity of GCGR ASOs targeting an 8X multiple repeat sequence

**Figure 1:**
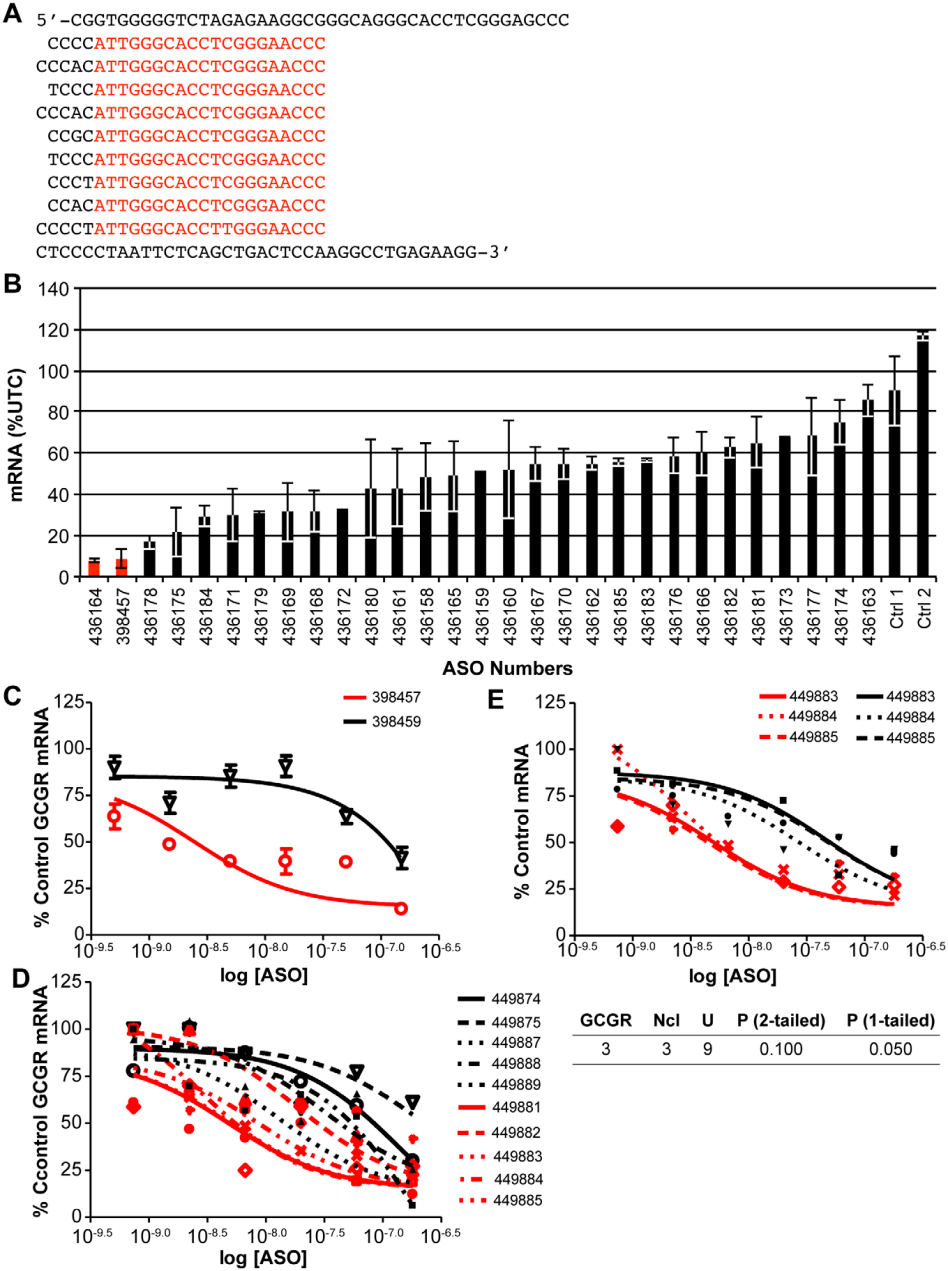
ASOs targeting a repeat sequence in *GCCR* are more active than ASOs targeting other regions of the gene. A) Sequence of part of *GCGR* intron 1. The first row shows the sequence of the intron 5′ of the repeats. Regions containing repeats (shown in red) are aligned in subsequent rows. A ninth repeat is homologous at 19/20 bases. Sequence of the intron 3′ of the repeats is shown in the bottom row. B) ASOs complementary to the repeat regions and other sequences in intron 1 were tested for ability to reduce levels of *GCGR* mRNA. Reduction was assessed by qRT/PCR 24 hours after electroporation of HepG2 cells in the presence of 1 µM ASO. Data were normalized to total RNA as measured by Ribogreen assay and are presented as expression in target mRNA relative to mock transfected control (UTC) for duplicate treatments with error bars representing range of activity. ASOs C1 and C2 do not have any perfect antisense alignment to human genes. C) Concentration response of multiple-site (398457) and single-site (395459) ASOs. HepG2 cells were treated with ASOs at concentrations between 0.5 nM and 150 nM in the presence of cationic lipid. *GCGR* mRNA reduction was assessed by qRT/PCR the following day. D) Concentration response curves of 17-nucleotide gapmer ASOs. *GCGR* mRNA reduction was assessed following treatment with ASO as detailed above. Red lines correspond to ASOs targeting multiply repeated sites; black lines to ASOs targeting single sites. E) Activity is increased when sites are repeated compared to activity on a transcript with the same single site. HepG2 cells were treated with 17-nucleotide ASOs at concentrations between 0.5 nM and 150 nM. *GCGR* (red lines) and *nucleoporin* (black lines) mRNA reduction was assessed by qRT/PCR the following day. Significance of the difference in IC_50_ values between ASOs targeting the GCGR multiple site *nucleoporin* single site as evaluated by Mann-Whitney U Test is shown below the figure.

It has previously been demonstrated that ASOs specific to the *glucagon receptor (GCGR)* transcript reduce its expression in liver improving hyperglycemia and hyperlipidemia [28]. Within intron 1 of GCGR the sequence 5′-ATTGGGCACCTCGGGAACCC-3′, is repeated eight times (Figure 1A). However, this 20 nucleotide sequence is not found elsewhere in the genome. In an attempt to identify more potent antisense inhibitors of human *GCGR*, a series of ASOs 20 nucleotides in length complementary to the repeated sequence as well as single sites (Table S3) were evaluated for the ability to reduce the target mRNA. All ASOs were targeted to sequences within the same intron, so contributions to activity related to rates of pre-mRNA processing and nuclear retention would be consistent for each ASO. Multiple site ASO 398457 is perfectly complementary to the repeat sequence, whereas ASO 436164 is shifted one nucleotide 5′ and is thus complementary to seven of the eight perfect repeats (Figure S1). All ASOs were “gapmers” with a core of DNA flanked by 2′-O-methoxyethyl (MOE) modified nucleotides; backbones were phosphorothioate. HepG2 Cells were electroporated in the presence of 1000 nM ASO as detailed in Materials and Methods with subsequent reduction of *GCGR* mRNA evaluated by qRT/PCR. The two ASOs targeting the repeated sites (red bars) were more active than any of the ASOs targeting single copy sites with intron 1 (Figure 1B). To expand and confirm this observation, HepG2 cells were next transfected with lipid-formulated repeat-targeting ASO 398457 or single-site targeting ASO 398459 at concentrations between 0.5 nM and 150 nM. For the multi-site ASO, the IC_50_ was approximately ∼4 nM (Figure 1C). In contrast, the IC_50_ for the ASO targeting a single site was greater than 100 nM. In other experiments, even the most potent ASOs targeted to single sites were at least 3-fold less potent than those targeting repeated sequence (data not shown), suggesting that by targeting repeated sites, a significant increase in potency can be achieved relative to even the best single-site targeting ASOs evaluated.

To optimize ASO activity, we designed 17-nucleotide MOE gapmer ASOs (Table S3) to the *GCGR* repeat sequence as well as single sites within the intron. HepG2 cells were lipid-transfected with ASOs at concentrations between 0.5 nM and 150 nM and levels of *GCGR* mRNA were determined. ASOs targeting the repeat site (449881-5) had IC_50_’s ranging between 3 and 19 nM, with an average IC_50_ of 10.6±5.8 nM (Figure 1D, red). In general ASOs targeting single sites (black lines) were less active with IC_50_’s ranging from 13 to >150 nM, and an average IC_50_ of 117.1±90.2. Although the difference in activity between 17-mer ASOs targeting the repeated site and single sites was not statistically significant (P = 0.0586), most likely as a result of the low levels of GCGR mRNA expression in HepG2 cells, the data suggest that targeting multiply repeated sites may result in increased ASO potency.

While the 20-mer repeat sequence was unique to GCGR, the 17 nucleotide sequence targeted by ASOs 449883–449885 also occurs once in the mRNA encoding nucleoporin. Interestingly, the IC_50_s for reduction of nucleoporin mRNA by these ASOs (average IC_50_ of 41.0±11.9 nM, Figure 1E, black lines) were similar to those observed for the ASOs targeting single sites in *GCGR*; whereas the average IC_50_ for the same ASOs targeting the *GCGR* repeated sequence was 3.5 nM±0.9 (Figure 1E, red lines). These data again suggest that targeting repeated sites may result in increased ASO potency relative to the same site present only once in a target.

### Targeting repeated regions leads to increased antisense oligonucleotide potency

To further explore whether ASOs targeting repeated sites in an mRNA leads to an increase in ASO activity and potency in other targets, we conducted an analysis of primary transcripts from 39787 genes. Surprisingly, we found that repeated sequences unique to a gene are relatively common in the human transcriptome; approximately 13% of human genes have regions of at least 16 nucleotides that are unique to the gene repeated three times or more and many more have two repeats (Table 1). These repeated sequences are more commonly found in introns than exons; 38% of primary transcripts have at least two repeats, whereas 9% of processed transcripts possessed two repeats or more. The majority of both pre-mRNAs and mature mRNAs containing these repeats are protein coding (Figure S2 A/B). Repeated sequences were identified with nearly equal abundance in the CDS and 3′ UTR of mRNAs; however, repeats were fairly rare in the 5′ UTR (Figures S2 C–E).

**Table 1:** Identification of 16-nucleotide repeat sequences unique to single genes in the human transcriptome.

Repeats	Primary	Processed
**Total**	39787 (100)	27638 (100)
**>/ = 2**	15264 (38.4)	2559 (9.3)
**>/ = 3**	5203 (13.1)	625 (2.3)
**>/ = 4**	3338 (8.4)	360 (1.3)
**>/ = 5**	2605 (6.6)	249 (0.9)

The total number of primary and processed transcripts with the given number of repeats unique to a single transcript is shown. Numbers in parentheses indicate the percent of the total transcripts containing a given number of unique repeat sequences.

**Figure 2:**
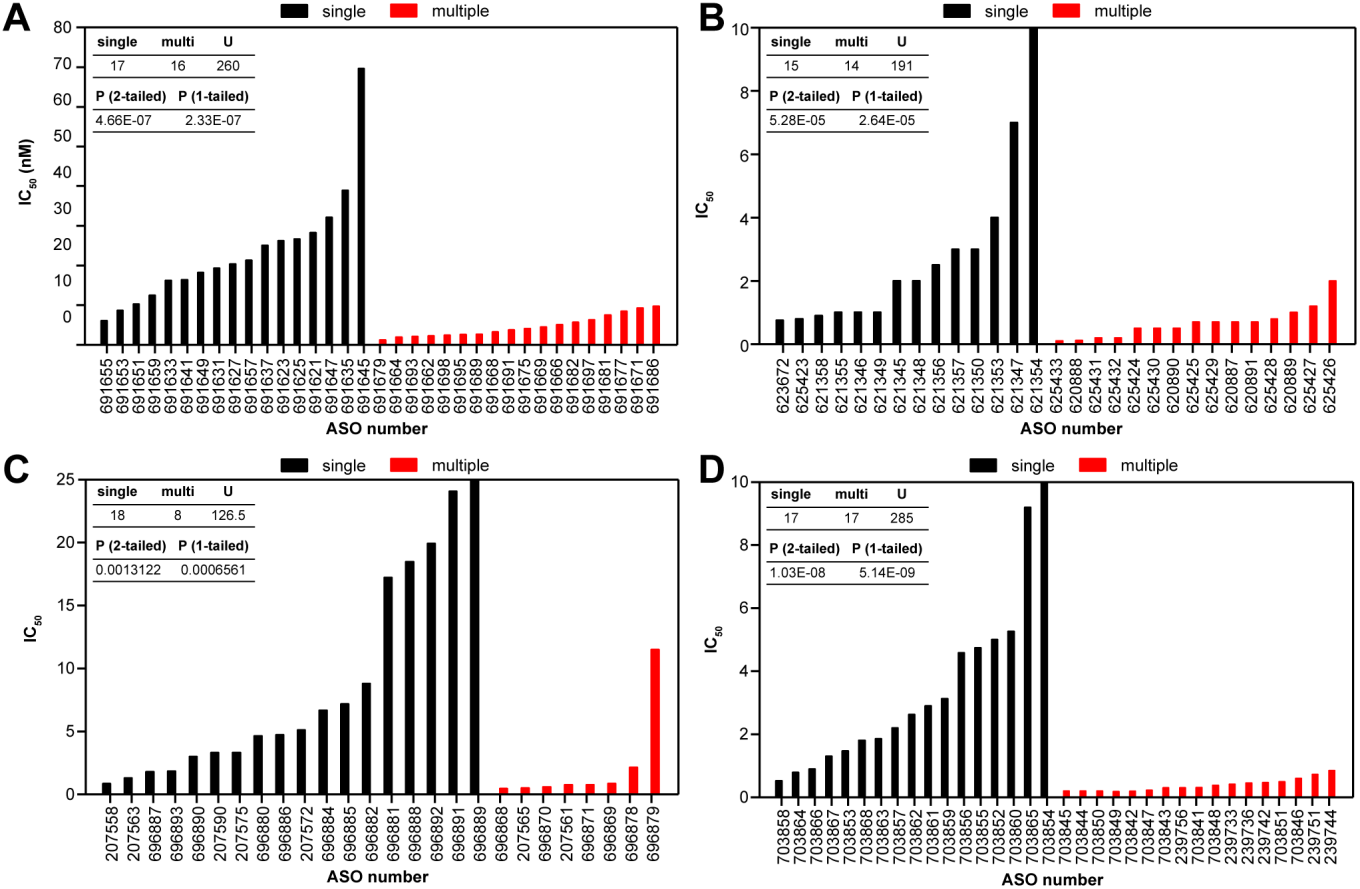
ASOs targeting repeated regions are more active than those targeting single sites. A) HeLa cells were lipid-transfected with 16-mer cEt gap-mer ASOs targeting *STAT3* intron 6 at concentrations between 0.3 and 50 nM. *STAT3* mRNA reduction was assessed by qRT/PCR the following day. IC_50_s were calculated from concentration response curves using GraphPad PRISM software and significant difference in IC_50_ values between all ASOs targeting single sites (black) and those targeting multiple sites (red) calculated using the Mann–Whitney U test (inset) B) ASO activity is enhanced at low copy repeats. HeLa cells were lipid transfected with 18-mer MOE gap-mer ASOs at concentrations between 0.3 and 50 nM with ASOs targeting single sites or two-copy repeat sites in MAPT intron 7. MAPT mRNA reduction was assessed by qRT/PCR the following day. IC_50_s were calculated from concentration response curves as for *STAT3*. C) ASOs targeting *OGFR* exonic repeats are more active than those targeting single sites. HeLa cells were lipid transfected with 20-mer MOE gap-mer ASOs at concentrations from 0.1 to 30 nM with ASOs targeting single sites or multiply repeated sites in the coding sequence of OGFR. OGFR mRNA reduction was assessed by qRT/PCR the following day and IC_50_s calculated and analyzed as above. D) ASO activity is higher when repeats rather than single sites are targeted in the 3′ UTR of *BOK*. HeLa cells were lipid-transfected with 20-mer MOE gap-mer ASOs at concentrations from 0.1 to 100 nM. *BOK* mRNA reduction was assessed by qRT/PCR the following day. IC_50_s were calculated from concentration response curves as above.

Within a 696-nucleotide span of intron 6 of STAT3, there are 36 16-nucleotide sequences repeated between eight and 36 times (Figure S3). 3-10-3 constrained ethyl (cEt) gapmer ASOs were designed to these multiply repeated sites and to an equal number of 16-mer single sites within the same intron (Table S4). cEt gapmer ASOs were used rather than 2′ MOE gapmers because they have greater affinity for the target RNA thus allowing for comparable binding with shorter ASOs [23]. HeLa cells were lipid transfected with ASOs at concentrations between 0.3 and 50 nM. Reduction of *STAT3* was assessed by qRT/PCR the following day. IC_50_s for single-site ASOs varied from 2 to 70 nM (Figures 2A &, S3B black). ASOs targeting repeated sites had IC_50_s ranging from 1 to 10 nM (red). For single sites the average IC_50_ was determined to be ∼21 nM, while for ASOs targeting repeat sites, the average IC_50_ of 4.5 nM was significantly less (P<0.001). Interestingly, the most potent ASOs were not necessarily those complementary to the sequences repeated most often. However, many of the ASOs perfectly complementary to sites repeated eight or nine times have single mismatches to many more. It could also be the case that the maximum effect on potency plateaus after a certain number of repeats and that additional repeats give no advantage.

We next wanted to determine whether ASO activity was enhanced even when there were only two copies of a target sequence in a gene. Several two-copy repeats were identified in introns 2, 7, and 10 of the pre-mRNA encoding microtubule-associated protein tau (*MAPT*). ASOs were designed to both single sites and repeat sites in each of these introns (Table S5). Significant activity was observed only for ASOs targeting sequences in intron 7. This seems to indicate that activity is dependent on the processing kinetics of a particular intron, as previous studies have suggested that introns spliced more slowly are most susceptible to ASO targeting [16]. It is also possible that differences in secondary or tertiary RNA structure between the introns may influence ASO activity [15]. Next, HeLa cells were treated with multi-site and single-site intron 7 ASOs (Figure S4) at concentrations from 0.1 to 100 nM. Reduction of *MAPT* was accessed by qRT/PCR the following day. IC_50_s for single-site ASOs varied between 0.8 and 27 nM, with an average IC_50_ of 4 nM (Figure 2B, black bars). In contrast, ASOs targeting repeated sites had IC_50_s from 0.1 to 2 nM, with an average IC_50_ of 0.7 nM (red bars). These data indicate that even when targeting only two repeats, there is a significant improvement in ASO potency relative to single target sites (P<0.001).

We next evaluated ASO targeting of repeated sequences present in exons. Although unique repeats are rarer in exons than introns, activity of ASOs in exonic repeats should be less dependent on pre-mRNA processing kinetics than activity in introns. The coding sequence of the human *opioid growth factor receptor* (*OGFR*) gene contains a polymorphic region of six imperfect 60-nucleotide tandem repeats (Figure S5). HeLa cells were lipid-transfected with multi-site and single-site ASOs (Table S6) at concentrations from 0.1 to 30 nM. Reduction of *OGFR* mRNA was determined by qRT/PCR. ASOs targeting single sites had a broad range of IC_50_s (from 0.9 to 42.9 nM) and an average IC_50_ of 9.7 nM (Figure 2C & S5B, black). ASOs targeting the repeated sequence were significantly more active (P<0.001), with an average IC_50_ of 2.2 nM and a range from 0.5 to 11.5 nM (red). Because the *OGFR* repeats are imperfect, the ASOs screened were perfectly matched to between three and six sites. It is interesting to note that the two least active ASOs targeting repeats, 696878 and 696879, had only three perfect matches within the exon, whereas the rest had five or six matches. If only these ASOs with five or six perfect matches are evaluated, the average IC_50_ improves to 0.7 nM, making these multi-site targeting ASOs on average almost 14-fold more potent than those targeting single sites.

Another gene, *Bcl-2-related ovarian killer* (*BOK*), contains a 41-nucleotide sequence that is imperfectly repeated 15 times in the 3′ UTR. Several ASOs were designed to target portions of the repeat sequence as well as single sites on the mRNA and were evaluated for activity at concentrations ranging from 0.1 to 100 nM (Table S7, Figure S6). As observed with other targets, the most potent ASOs were those targeting repeated sequence regions (Figure 2D, black bars). Single-site ASOs to this target were, in general, very potent as well, with an average IC_50_ for reduction of target mRNA of 3.6 nM; however, those ASOs designed to target the repeats had potencies approximately 10-fold better, with an average IC_50_ of 0.38 nM (red bars). In contrast to our observations for *OGFR*, for *BOK* there appeared to be little correlation between the number of repeats complementary to an ASO and potency. For example, the three most potent ASOs tested targeting *BOK* were complementary to nine, five, and 10 repeats, whereas the three least potent multi-site ASOs were complementary to 14 or 15 repeats. These data are more similar to those obtained in our screen of ASOs targeting *STAT3* intron 7 and suggest that after a certain number of repeats, little advantage is gained with respect to potency.

### Repeat site targeting is also more effective in a minigene system

**Figure 3:**
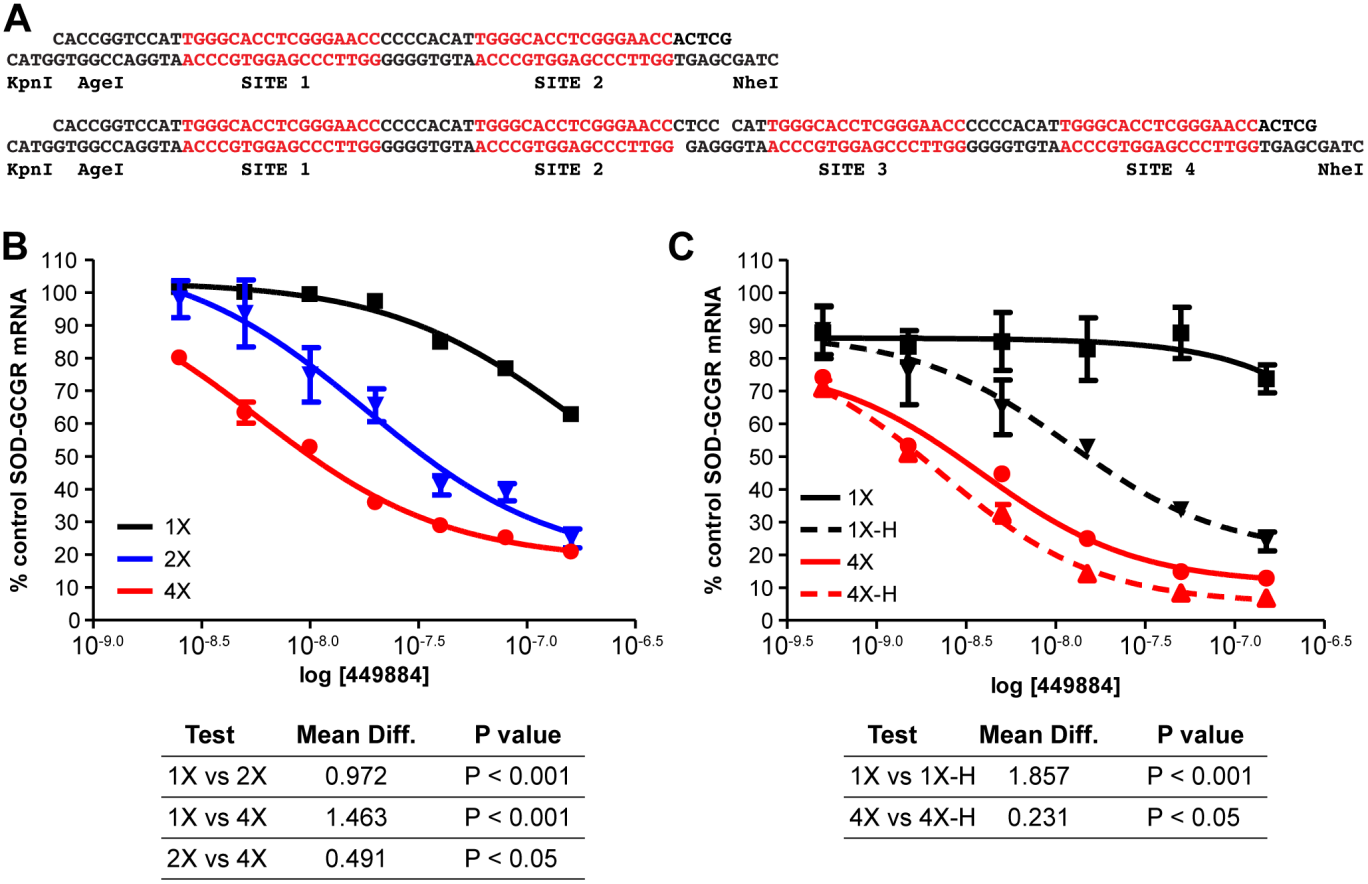
ASO activity is correlated with repeat number in a minigene system. The *GCGR* repeat sequence was inserted into the intron of a SOD1 minigene. A) Sequences of two- and four-repeat *GCGR* inserts with restrictions sites for directional cloning. B) T-REx-293 cells harboring SOD-GCGR minigene constructs containing one, two, or four GCGR repeat sequences were transfected with ASO 449884 at concentrations from 2.5 to 150 nM. Minigene mRNA reduction was assessed by qRT/PCR the following day. IC_50_ curves are plotted for single (black), two (blue), and four (red) repeat-containing constructs. Best-fit values for the logIC_50_ of the dose response curves were analyzed by one-way ANOVA and compared using Bonferroni’s multiple comparison test. C) Overexpression of RNase H results in significantly increased activity at the single GCGR site. *E. coli* RNase H was stably overexpressed in T-REx-293 cells harboring the SOD-GCGR minigene containing one or four repeat sequences. Minigene cell lines +/− *E. coli* RNase H were transfected with ASO 449884 at concentrations from 2.5 to 150 nM. Minigene mRNA reduction was assessed by qRT/PCR the following day. IC_50_ curves are plotted for single (black) or four (red) repeat sequences. Control cells were those that do not overexpress RNase H, solid lines; RNase H overexpressing cells, dashed lines. Significance was calculated as above.

To better understand the effects of targeting repeats on antisense activity, we cloned the *GCGR* repeat sequence into the intron of a SOD1 minigene construct [14] as a single site or as two or four tandem repeats (Figure 3A). Cells with stably integrated minigenes were treated with ASO 449884 at concentrations from 2.5–150 nM. Consistent with the results of the *MAPT* screen (Figures 2B & S4), ASO activity was significantly improved when the ASO was complementary to two repeats in the target RNA and a further increase activity relative to the single site was observed when four repeats were present in the target mRNA (Figure 3B).

If cellular levels of RNase H1 were not rate limiting, one would expect little additional activity at multiply repeated sites as compared to single sites. We therefore next determined activity for ASO 449884 targeting mRNA in single and multiple repeat minigene cell lines constitutively overexpressing *E. coli* RNase H [14]. In the absence of overexpressed RNase H, little degradation of the mRNA containing a single target in the intron was observed (Figure 3C, solid black line), whereas in the cell line with the four repeats the IC_50_ was approximately 4 nM (Figure 3C, red line). RNase H overexpression increased the degradation of the target with the single site significantly relative degradation with normal RNase H1 levels (Figure 3C, compare solid to dashed black lines). Consistent with our hypothesis, less of an increase was observed upon overexpression of RNase H in degradation of the target with four repeats (Figure 3C, compare solid to dashed red lines). These data also suggest that a maximal amount of activity can be reached, but not surpassed.

**Figure 4:**
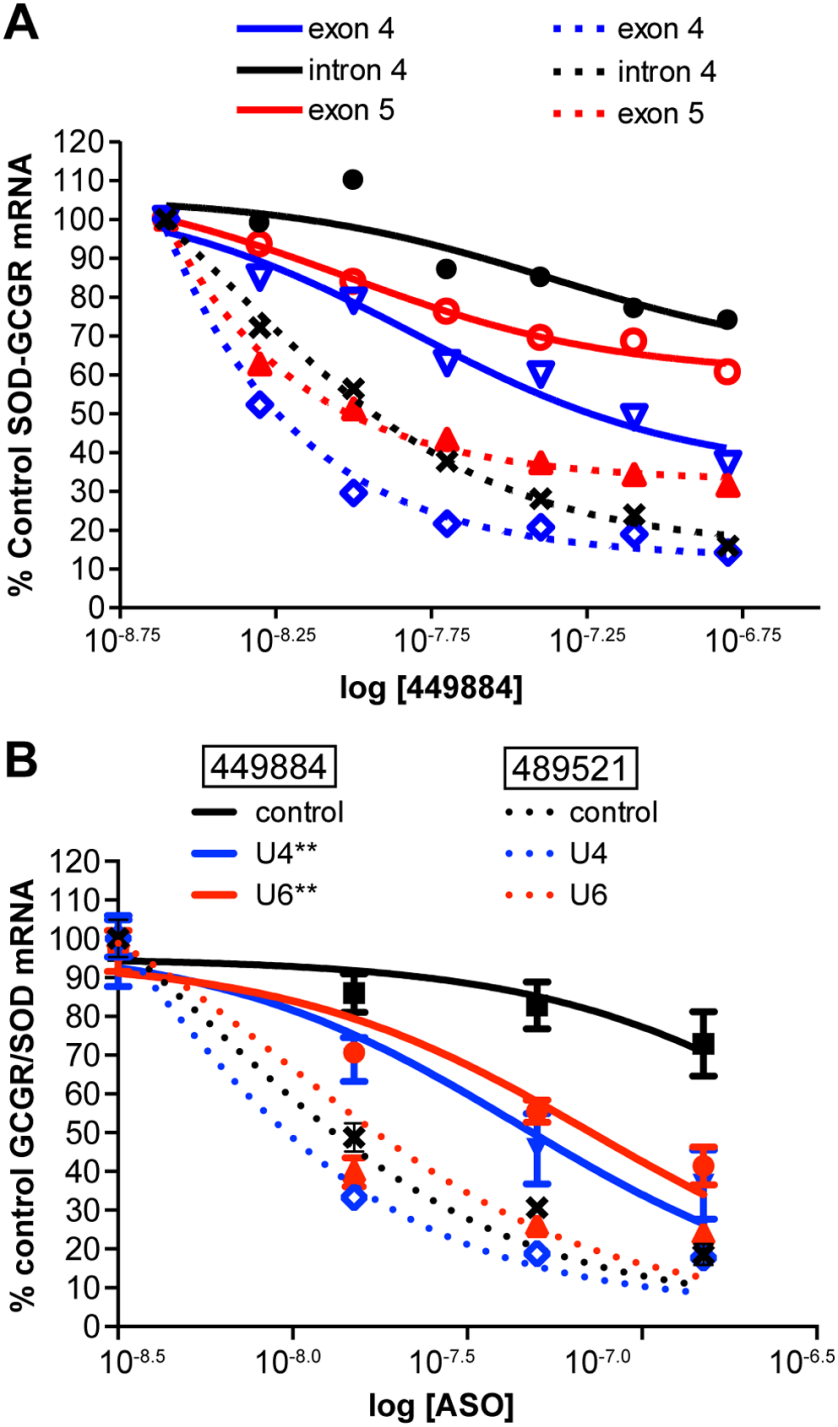
Effect of repeat localization and splicing rate on ASO activity. A) T-REx-293 cell lines harboring SOD-GCGR minigene constructs containing one (solid lines) or four (dashed lines) repeat sequences in exon 4 (blue), intron 4 (black), or exon 5 (red) of SOD1 were transfected with ASO 449884 at concentrations from 2.5 to 150 nM. Minigene mRNA reduction was assessed by qRT/PCR after 8 hours. IC_50_ curves are shown. B) The SOD-GCGR minigene construct containing a single repeat sequence in SOD1 intron 4 was treated with ASO complementary to U4 or U6 snRNAs. After 24 hours cells were transfected with ASO 449884 or with control ASO 489521 at 15, 50, and 150 nM. Minigene mRNA reduction was assessed by qRT/PCR. Results are presented as percent mock-transfected control. Dashed lines, ASO 489521; solid lines, ASO 449884. Black, control cells with no snRNA reduction; red, U4 snRNA reduced; blue U6 snRNA reduced. (**, P<0.05 by one-way ANOVA of best-fit values for the logIC_50_ and Newman-Keuls multiple comparison test of activity in control vs snRNA reduced cells).

We next sought to determine whether the position of the repeat within the pre-mRNA affects activity at multiply repeated sites. One or four of the *GCGR* repeat sequences were cloned into the SOD1 minigene in either exon 4 or 5. These GCGR/SOD hybrid minigenes were stably integrated into T-REx-293 cells. These cells and cells with the minigene containing the repeat in the intron were treated with ASO 449884 at concentrations ranging from 5 to 100 nM. As a single site, very little activity was observed when the site was contained within the intron; more activity was observed with minigene cell lines containing the repeat sequence in either exon (Figure 4A, solid lines). These data suggest that for a weak site, localization on the transcript can influence activity possibly due to differences in secondary structure, RNA/protein interactions at or near the target site, or processing [14]. With four repeats of this rather weak site, ASO activity increased significantly, even within the intron (Figure 4A, dashed lines).

We have previously shown that treatment with RNase H1ASOs targeting U1 or U4 snRNAs effectively reduces snRNA levels and significantly reduces the rate of splicing of an SMN2 minigene, resulting in increased levels of pre-mRNA relative to spliced mRNA [25]. To evaluate whether sequence context or rate of pre-mRNA processing is reponsible for the weaker activity in the intron, the cell line with the integrated GCGR/SOD hybrid with the single site in the intron was treated with ASOs targeting either U4 snRNA (479333) or U6 snRNA (479338). Both of these ASOs effectively reduced the targeted snRNAs and significantly slowed processing of the SOD minigene as measured by accumulation of pre-mRNA relative to spliced message (Figure S7). Following snRNA reduction, cells were treated with ASO 449884 to target the GCGR site in the intron or with ASO, 489521, which targets SOD1 exon 5. As previously observed, in cells with normal levels of snRNAs very little activity was observed when the site was located in the intron (Figure 4B, solid black lines), whereas the exon control ASO demonstrated good activity (dotted black lines). In contrast, reduction of either U4 snRNA (red lines) or U6 snRNA (blue lines) resulted in a significant increase in activity at the intron single site, but had no effect on the activity of the exon control ASO.

### All GCGR 449884 repeat sites appear to be equally accessible to human RNase H1

**Figure 5:**
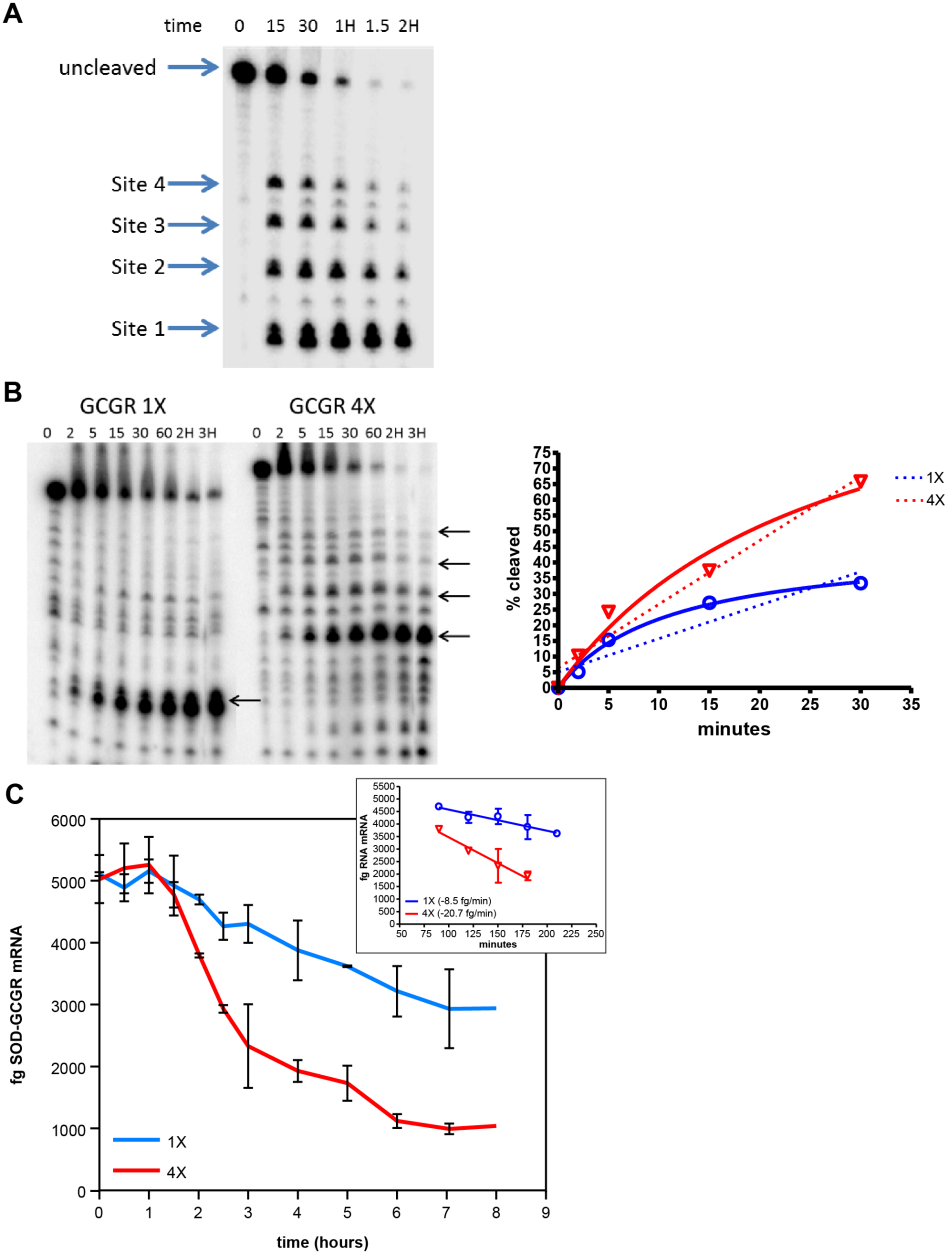
Human RNase H1 cleavage is more rapid when substrate contains multiple repeats of a target sequence than a single target. RNAs containing either a single or four *GCGR* repeat sequences were generated by T7 polymerase from PCR templates. The RNAs were dephosphorylated and end labeled. A) A 20-fold molar excess ASO 449884 was added to RNA, human RNase H was added, and samples were incubated for the indicated times. Cleavage products were visualized by electrophoresis on a denaturing polyacrylamide gel. B) A 4-fold molar excess of ASO 449884 was added to transcript containing one or four repeats. Following hybridization, RNase H cleavage assays were performed. Cleavage rates are plotted as the percent full-length RNA relative to that at 0 minutes for 1X RNA (blue) or 4X RNA (red). Non linear fit of data, solid lines; linear regression of data, dashed lines. C) Transcription of SOD-GCGR minigene constructs containing one or four repeat sequences was induced by addition of TET. After removal of TET, cells were transfected with ASO 449884 at 50 nM. ASO treatment was terminated, and RNA purified at the indicated times. Minigene RNA reduction was assessed by qRT/PCR with standards of known quantity for 1X RNA (blue) and 4X RNA (red). Data are plotted as fg mingene RNA vs. time; linear regression analysis (inset).

To determine the biochemical mechanism responsible for greater RNase H1 cleavage of repeated regions, *in vitro* RNase H1 cleavage reactions were performed using end-labeled RNA containing one or four of the *GCGR* repeat sequences. The RNA with four repeat sites was incubated with a 20-fold molar excess of ASO 449884, human RNase H was added, and the reaction was incubated at 37°C for the indicated time. Cleavage products were resolved on denaturing acrylamide gels. At early time points, all four sites appeared to be cleaved at equal rates (Figure 5A, compare band intensities at 15 minutes). However, as the reaction proceeded, the substrate was processed to the smallest labeled fragment, presumably as a result of multiple cleavages of each RNA.

### Human RNase H1 cleaves substrate with multiple target sites more rapidly than substrate with a single site

We next compared the cleavage rates of transcripts with either a single repeat sequence (1X RNA) or four repeat sequences (4X RNA). End-labeled RNA (0.1 pmol) was hybridized with 40 pmoles of ASO 449884. The reaction was incubated with human RNase H at 37°C for the indicated times. Cleavage fragments were resolved on denaturing acrylamide gels (Figure 5B). The rate of cleavage as measured by disappearance of the full-length RNA was significantly faster for the 4X (red) than the 1X (blue) RNA transcript. Similar results were observed in cells. Transcription of SOD/GCGR 1X or 4X RNA was induced overnight with tetracycline (TET). The next morning cells were washed to remove TET, then transfected with 50 nM ASO 449884. Cells were harvested at various times following initiation of ASO treatment, and RNA reduction was assayed by qRT/PCR. Target RNA reduction was not observed until 1.5 to 2 hours after the initiation of transfection, suggesting that this is the time required for the ASO to traverse the cell, scan available transcripts, bind to a cognate site, recruit RNase H1, and induce a measurable decrease in target RNA. For the cell line that expresses the transcript with the four repeat sequences, the rate of degradation was approximately 3-fold faster than for the transcript with a single site (Figure 5C).

**Figure 6:**
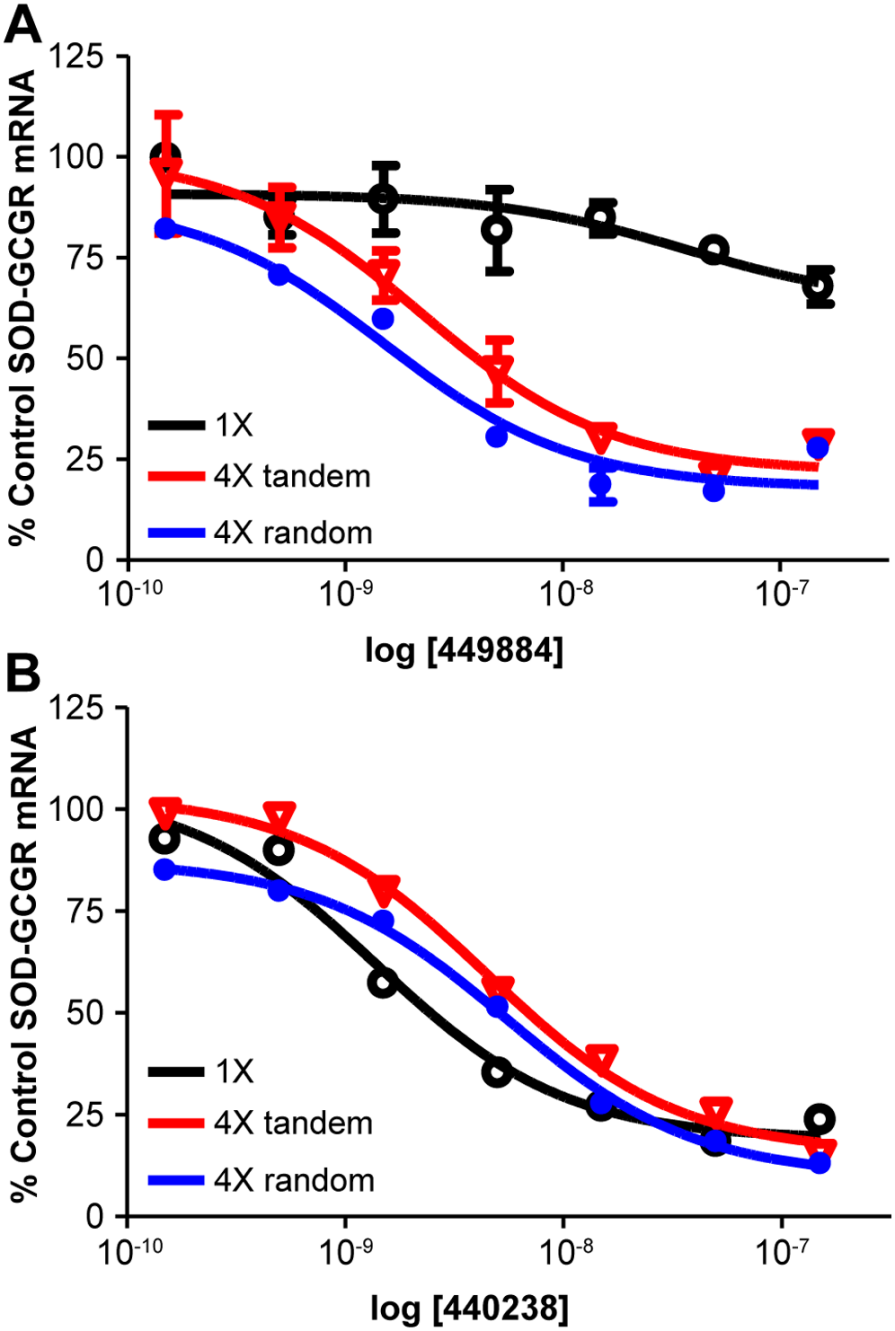
Repeats are not required to be in tandem for enhanced ASO activity. Four *GCGR* repeats were interspersed throughout in the SOD1 minigene by site-directed mutagenesis. T-REx-293 cell lines harboring SOD-GCGR minigene constructs containing single (black), four tandem (red), or four dispersed repeats (blue) were treated with either ASO 449884 or ASO 440238 at 0.3 to 100 nM. Minigene mRNA reduction was assessed by qRT/PCR after 8 hours. A) IC_50_ for repeat-targeting 449884. B) IC_50_ for SOD1 exon-targeting 440238.

To determine whether multiple repeats are required to be in tandem for enhanced ASO activity, we used site directed mutagenesis to insert the *GCGR* repeat at four sites dispersed throughout the SOD1 minigene (positions 19, 91, 472, and 523). Stable cell lines harboring minigenes with four tandem repeats in exon 5 or four non-tandem sites were treated with ASO 449884 (Figure 6A). As a control for transfection variability between cell lines, cells were also treated with ASO 440238, which targets a single site in exon 5 of each minigene. The presence of either tandem and non-tandem repeats resulted in similar increases in ASO potency relative to the same site present once in the minigene (Figure 6B).

## Discussion

Numerous factors affect the potency and specificity of ASOs in cells including biostability, cellular uptake, subcellular distribution, protein interactions, and hybridization affinities for the target RNA [29]. Here, we sought to evaluate the effect of targeting repeated sequences on activity. Our evaluation of almost 40,000 pre-mRNAs indicates that a significant proportion of both pre-mRNAs and spliced mRNAs harbor repeated sequences of at least 16 nucleotides that are unique to the particular gene (Table 1).

Although we and others have used ASOs or siRNAs to target genes containing short expanded repeats [30]–[32], these repeats are thought to form highly stable secondary structures and are often avoided when designing ASOs [33], [34]. Specific reduction has been observed in some cases when CAG repeats have been directly targeted [35], however, large doses of the ASO were required to produce significant activity in animals, which may lead to nonspecific downregulation of numerous other transcripts containing expanded CAG repeats. In this study we evaluated ASO-mediated reduction of RNA transcripts of five genes containing 16–20 nucleotide repeated sequences present only in the targeted gene. These targets had from two to 35 repeats in introns or in exons. In each case, targeting the repeated sequence led to significantly greater ASO potency than targeting a unique site within the same gene. These differences do not appear to be related to variations in ASO binding affinities as calculated Tm’s for single and multiple repeat targeting ASOs generally had similar values. When the repeated sites were located within exons, the most active ASO targeting a repeated sequence was far more active than the most active ASO targeting a non-repeated site (Figure 2C/2D). In introns, the results were more complicated (Figures 1, 2A/2B). For example, in the *MAPT* gene, there are several two-copy repeats in introns 2, 7, and 10, but only ASOs targeting repeat sequences in intron 7 were active. It has previously been shown that secondary structure can have significant effects on ASO hybridization and activity [14], [15]. Therefore, it is possible that the lack of observed activity in introns 2 and 10 may be due to differences in secondary or tertiary structures in the regions of the target sequences. More likely, differences in activity between introns result from differential rates of processing of the pre-mRNA in these regions due to disparities in splice site strength, intron length, or other unknown factors [16], [17]. Our data appear to confirm this. While the *GCGR* repeats are predicted to form reasonably stable stem-loop structures (Figure S1 B), ASO activity was improved in the 2X and 4X minigene constructs relative to the single site, which presumably is not as highly structured (Figure 3). Further, antisense activity against *GCGR* repeat sequences incorporated into the minigene intron confirmed that splicing can play a role in the activity of intron-targeting ASOs, as the level of ASO activity on the transcript with a single repeat sequence within the intron was increased by inhibition of pre-mRNA processing (Figure 4B). These data also indicate that a potential ASO binding site in an intron may be removed by the splicing machinery before the ASO finds its cognate site and RNase H cleaves the heteroduplex, whereas a site in which the rate of heteroduplex formation and RNase H1 recruitment is more rapid than the splicing rate can result in cleavage of the RNA.

In human cells RNase H1 is rate-limiting with respect to ASO activity. Increasing the levels of human RNase H1 in cells increases the potency of ASOs, whereas decreasing the levels of the enzyme leads to decreased ASO potency [4], [14]. In our minigene system, overexpression of RNase H had a significantly greater effect on ASO potency when the ASO targeted a single site in a transcript than when the transcript contained repeats of the target site (Figure 3C). This suggests that the increase in activity at repeated sites is the result of increasing the ASO hybridization frequency and recruitment of RNase H1 to a particular RNA target, which increases the rate of target cleavage and degradation (Figures 5B/5C). Thus, it does not appear that there is cooperativity between repeated regions. Furthermore, *in vitro* cleavage assays demonstrate that no single repeated site was preferred over another in a transcript containing tandem repeats (Figure 5A). Although all the endogenous genes we evaluated had repeats near each other on the transcript (from eight to 40 nucleotides apart), similar activities were observed when four repeat sequences were inserted tandemly in the minigene or widely spaced throughout (Figure 6). This result confirms that the mechanism underlying enhanced potency is likely simply increased rates of productive ASO/RNA interactions with RNase H1 rather than cooperativity induced by the repeated sequence.

In the highly controlled SOD/GCGR minigene system, there was a reasonable correlation between the number of repeats and potency: as the number of repeats was increased, the potency of ASO 449884 was also increased (Figure 3B). This increase in potency was independent of the localization of the repeat in the transcript (Figure 4A). In contrast, in endogenous RNAs, the correlation between the number of repeats and potency was far less convincing. For example, for multiply repeated sequences in intron1 of *GCGR*, there was over 10-fold difference in average potency between the ASOs targeting a sequence repeated eight times and the ASOs targeting a single site (Figure 1). In contrast, for ASOs targeting *STAT3*, the difference was only 4.7 fold despite the presence of up to 35 repeats (Figure 2A, S3). For ASOs targeting *MAPT* the difference (5.7 fold) was similar to those targeting *STAT3* despite the presence of only duplicate repeats in *MAPT* (Figure 2B, S4). This difference may be attributable to the chemistry of the ASOs used in these screens, as 16-mer cEt gapmers were used in *STAT3* experiments and 18-mer MOE gapmers were used in *MAPT* experiments. Further experiments will be required to determine weather the increased affinity of cEt relative to MOE gap-mers may have greater effects on potency at single sites as compared to multiply repeated sites. For exon-targeting ASOs (Figure 2C/2D), the difference in activity between repeat-targeting and single-site targeting ASOs was similar for *BOK* and *OGFR*, despite the fact that the former gene had twice the number of repeats. This suggests that other factors, such as RNA structure, protein binding, and splicing rate, may play important roles in determining the impact of repeats on ASO potency in endogenous RNAs.

ASOs have proven of value in experiments designed to determine gene functions and as therapeutic agents [36]. On an average mRNA or pre-mRNA, there are thousands of sites that can be targeted by a typical 20-nucleotide ASO. In practice, many oligonucleotides complementary to a transcript have little or no antisense activity. The challenge of antisense drug design is to identify the most active and potent compound as quickly and efficiently as possible. Many antisense design strategies have been employed in an attempt to produce more potent and specific antisense therapeutics and to reduce the time and cost of discovery [37]. Most of these strategies rely on algorithms to that take into account secondary structure, thermodynamic properties, gene features, and/or binding free energies to identify optimal sites in target mRNAs [38]–[40]; however, in our experience, use of these algorithms does not significantly reduce the need for *in vitro* screening of large numbers of compounds. The data presented in this manuscript clearly demonstrate that ASOs complementary to repeat sequences are more potent than ASOs targeting single sites in the same mRNA. Repeated sites are “hot spots” for ASO targeting, and the best ASOs targeting repeated sites more active than the best non-repeat-targeting ASOs. Clearly ASOs targeting repeats should match as few genes as possible. Even with our strict filtering, many targets were identified that contained unique repeated sequences.

Based on observations reported here and additional work reported in a number of recent publications [12], [14], [15], [18]–[20], we can now begin to construct a more satisfying intellectual framework with which to understand ASO activity. In summary, the critical factors that influence ASO activity and specificity identified to date are RNase H1 levels in the cell and the sequence preferences of the enzyme, RNA structure, subcellular localization of the RNA and the ASO, the position of the cognate site in the topology of the pre-RNA, the splicing rate (for intronic sites), proteins that bind to the RNA and compete with RNase H1 for the ASO duplex, affinity of the ASO for its target site, and the number of sites for the ASO in the target RNA. Incorporation of these parameters into antisense design algorithms should support more rapid and efficient identification of optimal ASOs. Equally importantly, these observations pertain not only to the desired activity, but also to off-target effects, so these data provide insights that support not just increased activity but also an increase in therapeutic index.

## Supporting Information

Figure S1 **GCGR intron 1 ASO screen.** A) The location of GCGR sites screened is represented relative to position on GCGR intron 1 (pink) for multiple repeat targeting ASOs (red) or single site ASOs (black). Bordering exons are shaded yellow. Alignments of each multiple repeat region screened were performed against the 20 fully resequenced individuals from the 1000 genomes project to verify that the repeat structures were fully conserved. B) Multiple repeat binding site structure prediction. The optimal secondary structure the first two repeats depicted in Figure 1A in dot-bracket notation with a minimum free energy of −15.70 kcal/mol was generated using RNAfold [41]. The location of the binding site and sequence for ASO 449885 is shown. Underlined bases are 2′MOE. Similar structures are formed by the other repeat pairs.
(PDF)

Figure S2 **Identification of repeated sequences unique to a gene.** Transcripts from 39787 genes were analyzed for 16-mer repeat sequences as detailed in Materials and Methods. A) Percentage of primary (pre-mRNA) or processed (spliced mRNA) transcripts harboring 2–5 16-mer repeats. B) The majority of genes with repeats encode protein. C) Distribution of repeated regions in CDS, 3′ UTR, and 5′ UTR. Shown is the number of repeats/gene for 2087 genes with repeats on processed transcripts having annotated CDS regions. D) Distribution of repeat regions in CDS, 3′ UTR, and 5′ UTR normalized by transcript region length for 2087 genes with repeats on processed transcripts having annotated CDS regions. E) Distribution of CDS, 3′ UTR, and 5′ UTR region as a function of length of processed transcript in genes with repeats.
(PDF)

Figure S3 ***STAT3*** **intron 6 ASO screen.** A) Location of *STAT3* ASO target sites. The location of sites screened is represented relative to position on *STAT3* intron 6 (pink) for multiple repeat targeting ASOs (red) or single site ASOs (black). Bordering exons are shaded yellow. B) IC_50_ curves for *STAT3* ASO screen using Exon 8/9 primer/probe set. Significant differences in IC_50_ values between all ASOs targeting single sites (black) and those targeting multiple sites (red) calculated using the Mann–Whitney U test is shown. C) IC_50_ curves for *STAT3* ASO screen using Exon 3 primer/probe set. D) Comparison of IC_50_ values obtained using Exon 8/9 (solid bars) or exon 3 (hatched bars).
(PDF)

Figure S4 ***MAPT*** **intron 7 ASO screen.** A) Location of *MAPT* ASO target sites. The location of sites screened is represented relative to position on *MAPT* intron 7 (pink) for multiple repeat targeting ASOs (red) or single site ASOs (black). Bordering exons are shaded yellow. B) IC_50_ curves for *MAPT* ASO screen using Exon 13/14 primer/probe set. Significant differences in IC_50_ values between all ASOs targeting single sites (black) and those targeting multiple sites (red) calculated using the Mann–Whitney U test is shown.
(PDF)

Figure S5 **OGFR ASO screen.** A) Location of OGFR ASO target sites. The location of sites screened is represented relative to position on OGFR mRNA (NM_007346) for multiple repeat targeting ASOs (red) or single site ASOs (black). The location of the primer/probe set is shown in green with CDS in grey and exons in yellow. B) IC50 curves for OGFR ASO screen using Exon 6/7 primer/probe set. Significant differences in IC50 values between all ASOs targeting single sites (black) and those targeting multiple sites (red) calculated using the Mann–Whitney U test is shown.
(PDF)

Figure S6 **BOK ASO screen.** A) Location of BOK ASO target sites. The location of sites screened is represented relative to position on BOK mRNA (NM_032515) for multiple repeat targeting ASOs (red) or single site ASOs (black). The location of the primer/probe set is shown in green with CDS in grey and exons in yellow. B) IC50 curves for BOK ASO screen using Exon 5 primer/probe set. Significant differences in IC50 values between all ASOs targeting single sites (black) and those targeting multiple sites (red) calculated using the Mann–Whitney U test is shown.
(PDF)

Figure S7 **Effect of snRNA reduction on SOD1 minigene processing.** A) Sequence of ASOs trageting snRNAs. ASOs are phosphorothioate at each position with MOE-substituted bases underlined. B) Northern analysis of U4/U6 snRNA reduction was carried out as previously described [25]. C) Effects of snRNA reduction on SOD1 minigene processing. SOD/TO cells were treated with ASOs targeting SRFS and snRNAs U1, U2, U4, and U6. After 24 hours minigene expression was induced by addition of TET to the media for 2 hours. Levels of minigene spliced and pre-mRNA were assessed by qRT/PCR using primer/probe set described previously [14]. Data is plotted as percent expression relative to mock treated control (TET) for spliced mRNA (solid bars) and pre-mRNA (striped bars).
(PDF)

Table S1 **Sequences of primers/probes used for qRT/PCR.** For primers complementary to the minigene, vector sequence is in lower case.
(PDF)

Table S2 **Sequences of primers used for insertion of GCGR site at non-tandem positions in the SOD1 minigene.**
(PDF)

Table S3 **Sequences of ASOs complementary to GCGR.** All ASOs are phosphorothioate at each position with MOE-substituted bases underlined. The number of sites is equal to the number of times the ASO is perfectly matched to the target sequence. Tm is calculated for RNA/DNA heteroduplexes [42].
(PDF)

Table S4 **Sequences of ASOs complementary to STAT3.** All ASOs are phosphorothioate at each position with cEt-substituted bases underlined. The number of sites is equal to the number of times the ASO is perfectly matched to the target sequence.
(PDF)

Table S5 **Sequences of ASOs complementary to MAPT.** All ASOs are phosphorothioate at each position with MOE-substituted bases underlined. The number of sites is equal to the number of times the ASO is perfectly matched to the target sequence.
(PDF)

Table S6 **Sequences of ASOs complementary to OGFR.** All ASOs are phosphorothioate at each position with MOE-substituted bases underlined.
(PDF)

Table S7 **Sequences of ASOs complementary to BOK.** All ASOs are phosphorothioate at each position with MOE-substituted bases underlined.
(PDF)
