# Supplementary figures and images for: Targeting of Repeated Sequences Unique to a Gene Results in Significant Increases in Antisense Oligonucleotide Potency
Source: PLoS One. 2014 Oct 15;9(10):e110615. doi: 10.1371/journal.pone.0110615 (PMC4198294; doi:10.1371/journal.pone.0110615)

**Figure S1.** *GCGR* intron 1 ASO screen.

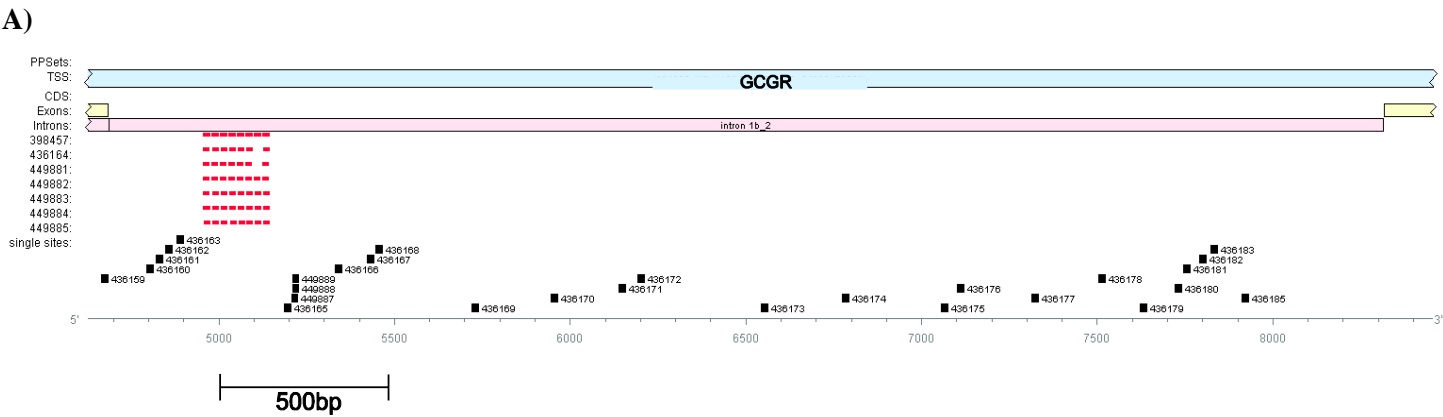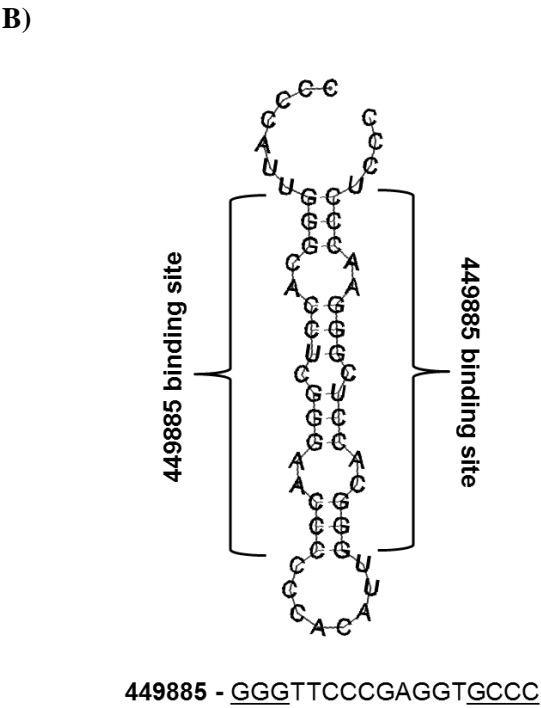

Supplement: Figure S1 — GCGR intron 1 ASO screen. A) The location of GCGR sites screened is represented relative to position on GCGR intron 1 (pink) for multiple repeat targeting ASOs (red) or single site ASOs (black). Bordering exons are shaded yellow. Alignments of each multiple repeat region screened were performed against the 20 fully resequenced individuals from the 1000 genomes project to verify that the repeat structures were fully conserved. B) Multiple repeat binding site structure prediction. The optimal secondary structure the first two repeats depicted in Figure 1A in dot-bracket notation with a minimum free energy of −15.70 kcal/mol was generated using RNAfold [41]. The location of the binding site and sequence for ASO 449885 is shown. Underlined bases are 2′MOE. Similar structures are formed by the other repeat pairs. (PDF) [file pone.0110615.s001.pdf]

**Figure S2.** Identification of repeated sequences unique to a gene.

A)

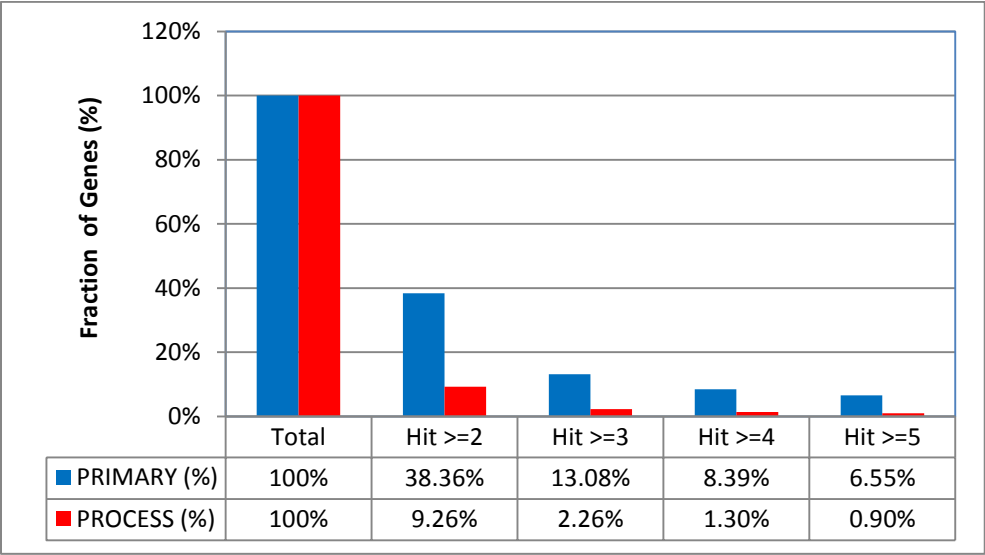

B)

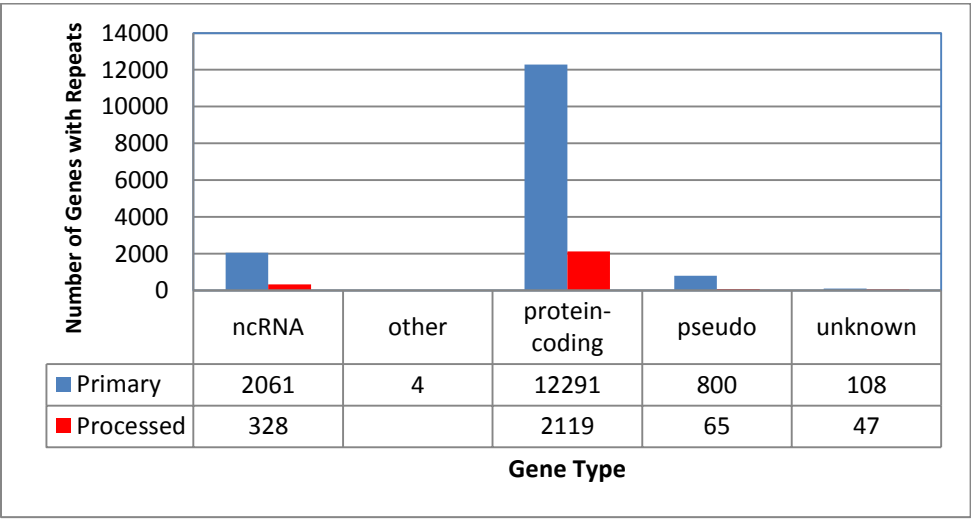

C)

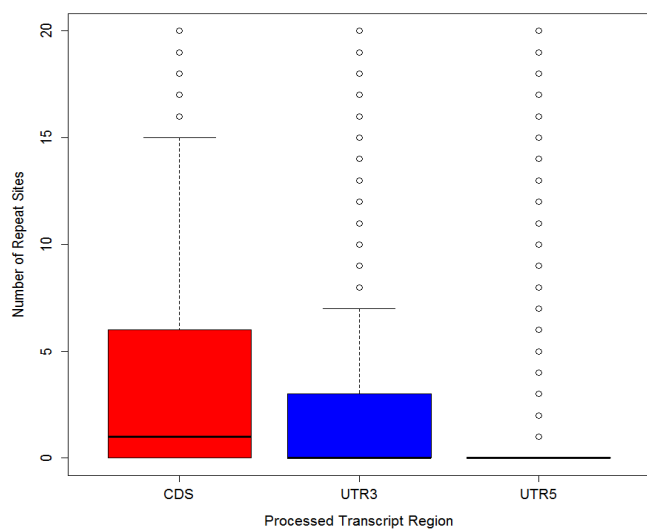

D)

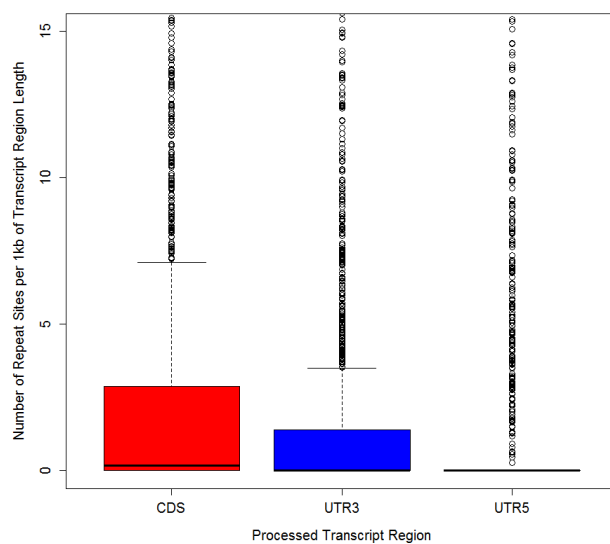

E)

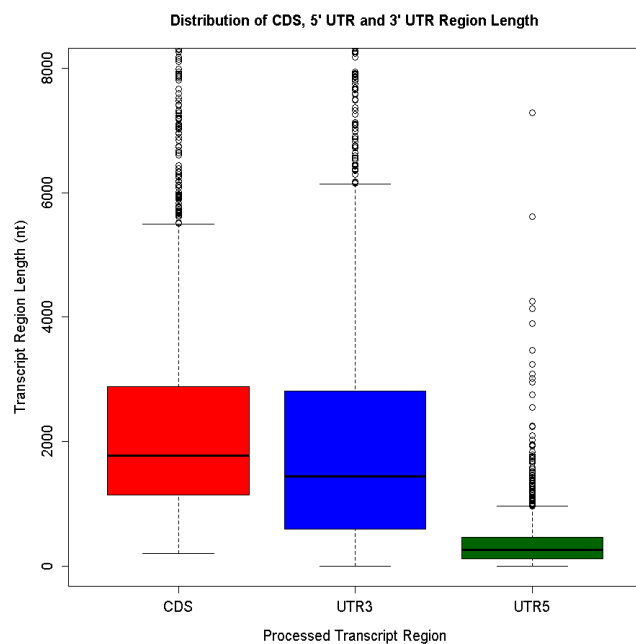

Supplement: Figure S2 — Identification of repeated sequences unique to a gene. Transcripts from 39787 genes were analyzed for 16-mer repeat sequences as detailed in Materials and Methods. A) Percentage of primary (pre-mRNA) or processed (spliced mRNA) transcripts harboring 2–5 16-mer repeats. B) The majority of genes with repeats encode protein. C) Distribution of repeated regions in CDS, 3′ UTR, and 5′ UTR. Shown is the number of repeats/gene for 2087 genes with repeats on processed transcripts having annotated CDS regions. D) Distribution of repeat regions in CDS, 3′ UTR, and 5′ UTR normalized by transcript region length for 2087 genes with repeats on processed transcripts having annotated CDS regions. E) Distribution of CDS, 3′ UTR, and 5′ UTR region as a function of length of processed transcript in genes with repeats. (PDF) [file pone.0110615.s002.pdf]

Figure S3. STAT3 intron 6 ASO screen.

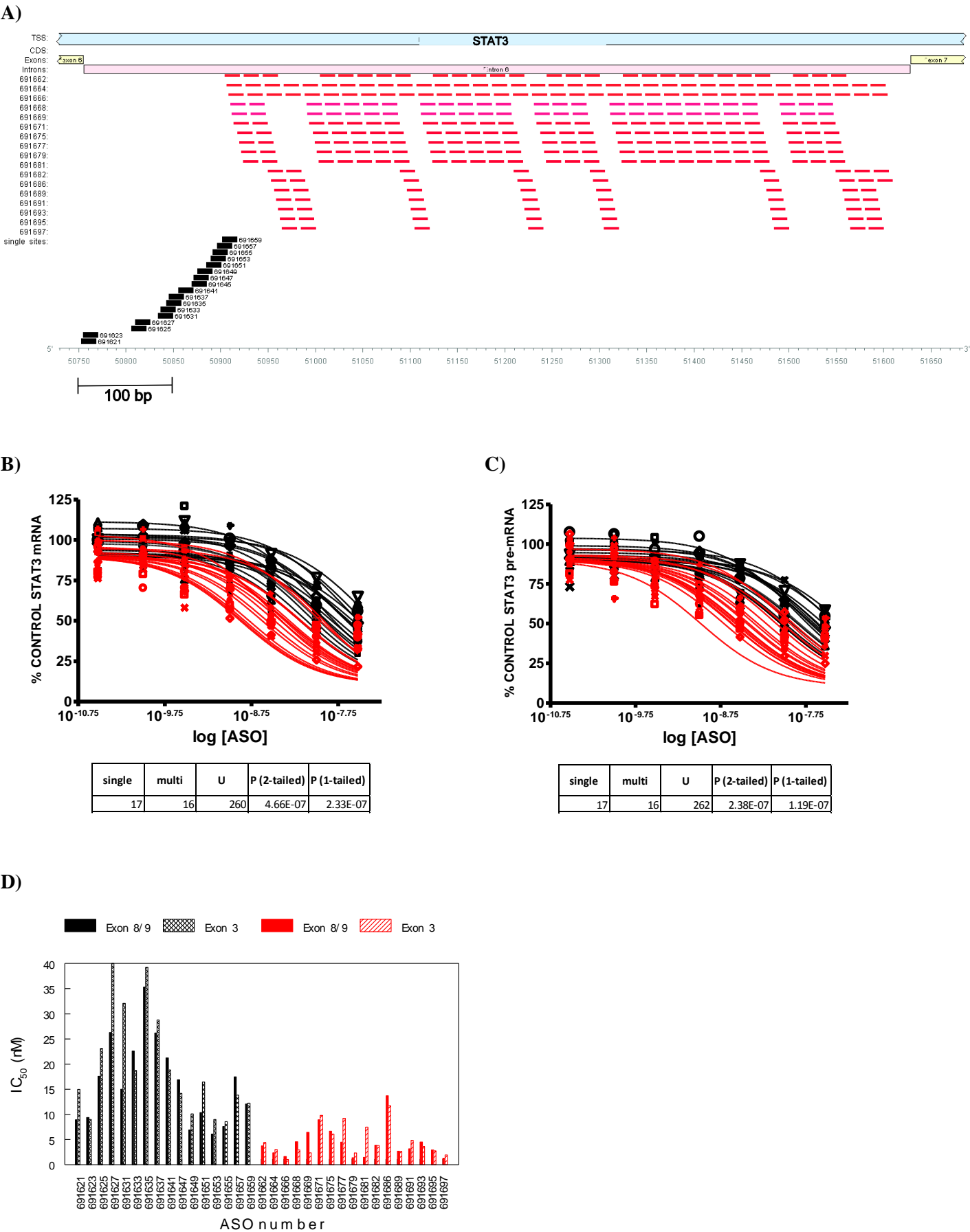

Supplement: Figure S3 — STAT3 intron 6 ASO screen. A) Location of STAT3 ASO target sites. The location of sites screened is represented relative to position on STAT3 intron 6 (pink) for multiple repeat targeting ASOs (red) or single site ASOs (black). Bordering exons are shaded yellow. B) IC50 curves for STAT3 ASO screen using Exon 8/9 primer/probe set. Significant differences in IC50 values between all ASOs targeting single sites (black) and those targeting multiple sites (red) calculated using the Mann–Whitney U test is shown. C) IC50 curves for STAT3 ASO screen using Exon 3 primer/probe set. D) Comparison of IC50 values obtained using Exon 8/9 (solid bars) or exon 3 (hatched bars). (PDF) [file pone.0110615.s003.pdf]

Figure S4. MAPT intron 7 ASO screen.

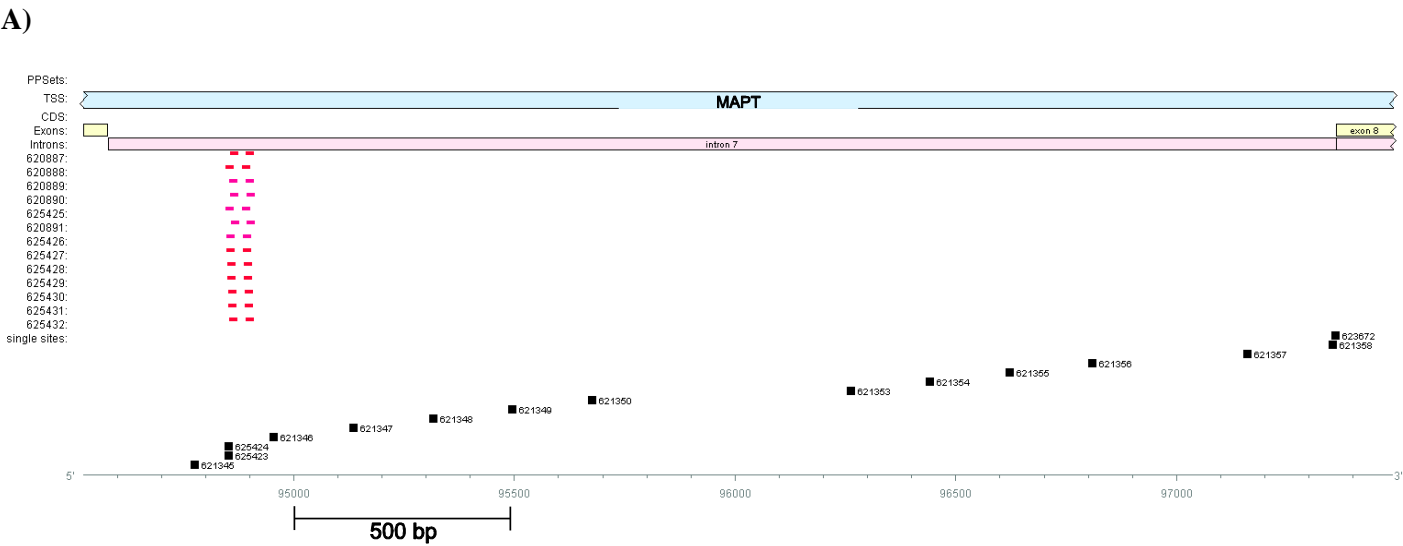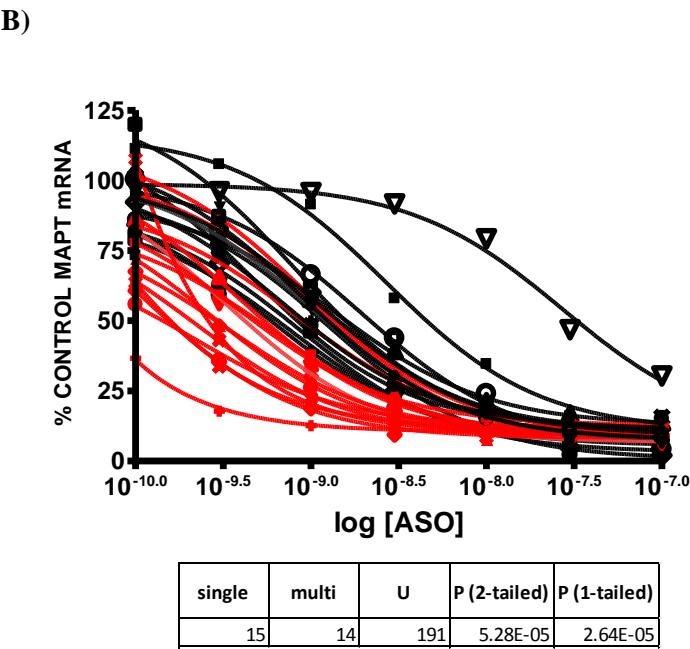

Supplement: Figure S4 — MAPT intron 7 ASO screen. A) Location of MAPT ASO target sites. The location of sites screened is represented relative to position on MAPT intron 7 (pink) for multiple repeat targeting ASOs (red) or single site ASOs (black). Bordering exons are shaded yellow. B) IC50 curves for MAPT ASO screen using Exon 13/14 primer/probe set. Significant differences in IC50 values between all ASOs targeting single sites (black) and those targeting multiple sites (red) calculated using the Mann–Whitney U test is shown. (PDF) [file pone.0110615.s004.pdf]

**A)**

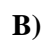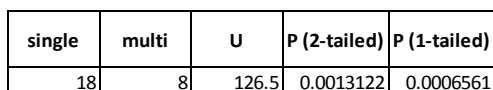

Supplement: Figure S5 — OGFR ASO screen. A) Location of OGFR ASO target sites. The location of sites screened is represented relative to position on OGFR mRNA (NM_007346) for multiple repeat targeting ASOs (red) or single site ASOs (black). The location of the primer/probe set is shown in green with CDS in grey and exons in yellow. B) IC50 curves for OGFR ASO screen using Exon 6/7 primer/probe set. Significant differences in IC50 values between all ASOs targeting single sites (black) and those targeting multiple sites (red) calculated using the Mann–Whitney U test is shown. (PDF) [file pone.0110615.s005.pdf]

Figure S6. *BOK* ASO screen.

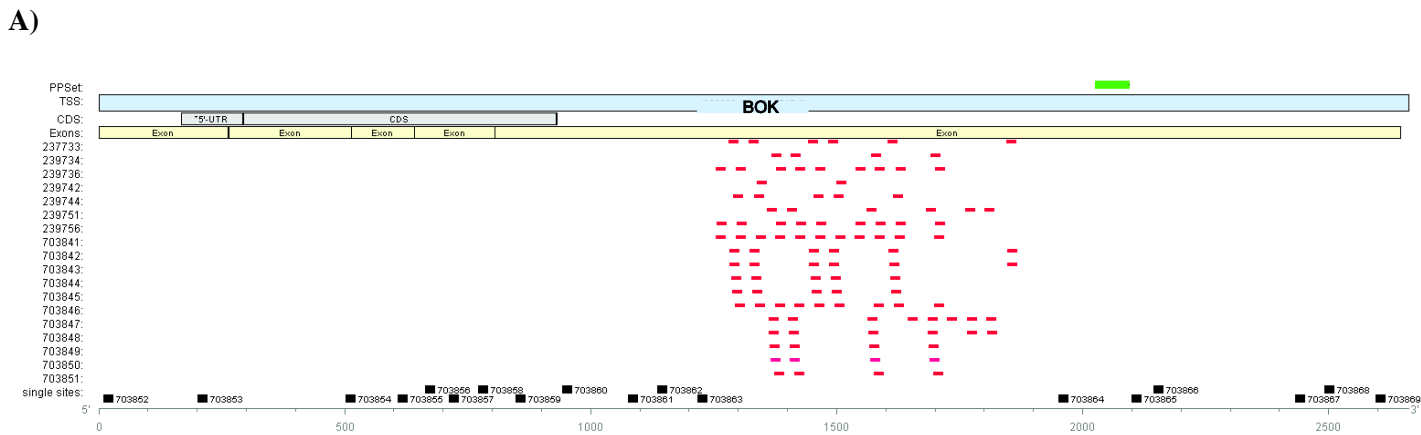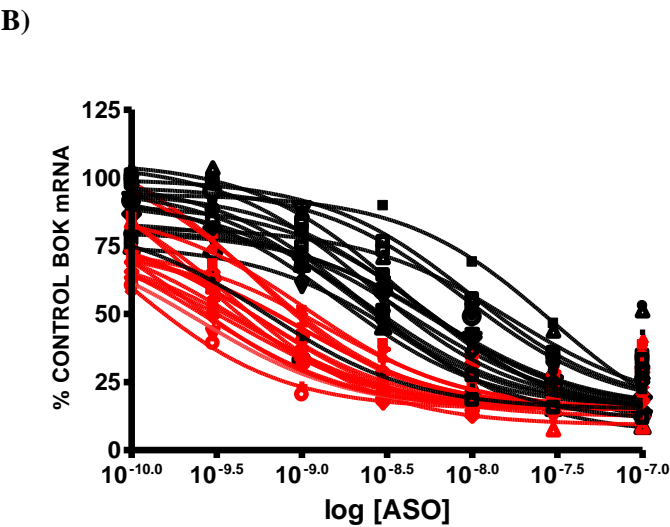

| single | multi | U   | P (2-tailed) | P (1-tailed) |
|--------|-------|-----|--------------|--------------|
| 17     | 17    | 285 | 1.03E-08     | 5.14E-09     |

Supplement: Figure S6 — BOK ASO screen. A) Location of BOK ASO target sites. The location of sites screened is represented relative to position on BOK mRNA (NM_032515) for multiple repeat targeting ASOs (red) or single site ASOs (black). The location of the primer/probe set is shown in green with CDS in grey and exons in yellow. B) IC50 curves for BOK ASO screen using Exon 5 primer/probe set. Significant differences in IC50 values between all ASOs targeting single sites (black) and those targeting multiple sites (red) calculated using the Mann–Whitney U test is shown. (PDF) [file pone.0110615.s006.pdf]
