# Supplementary material for: Targeting of Repeated Sequences Unique to a Gene Results in Significant Increases in Antisense Oligonucleotide Potency
Source: PLoS One. 2014 Oct 15;9(10):e110615. doi: 10.1371/journal.pone.0110615 (PMC4198294; doi:10.1371/journal.pone.0110615)

**Figure S7.** Effect of snRNA reduction on SOD1 minigene processing.

**A)**

| ISIS # | snRNA ASO Sequence | length | target |
|---|---|---|---|
| 479332 | TACTGCCACTGCGCAAAGCT | 20 | U4 |
| 479333 | GGTATTGGGAAAAGTTTTCA | 20 | U4 |
| 479338 | CCATGCTAATCTTCTCTGTA | 20 | U6 |
| 479339 | TTGCGTGTCATCCTTGCGCA | 20 | U6 |

**B)**

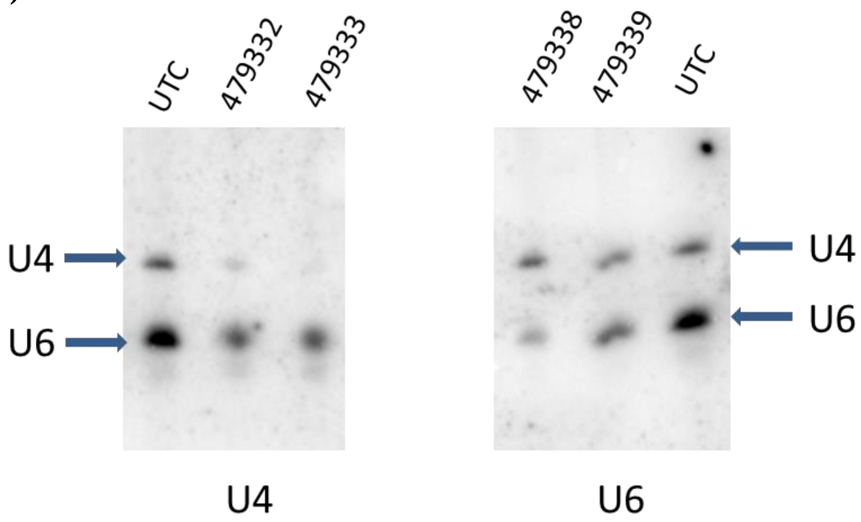


**C)**

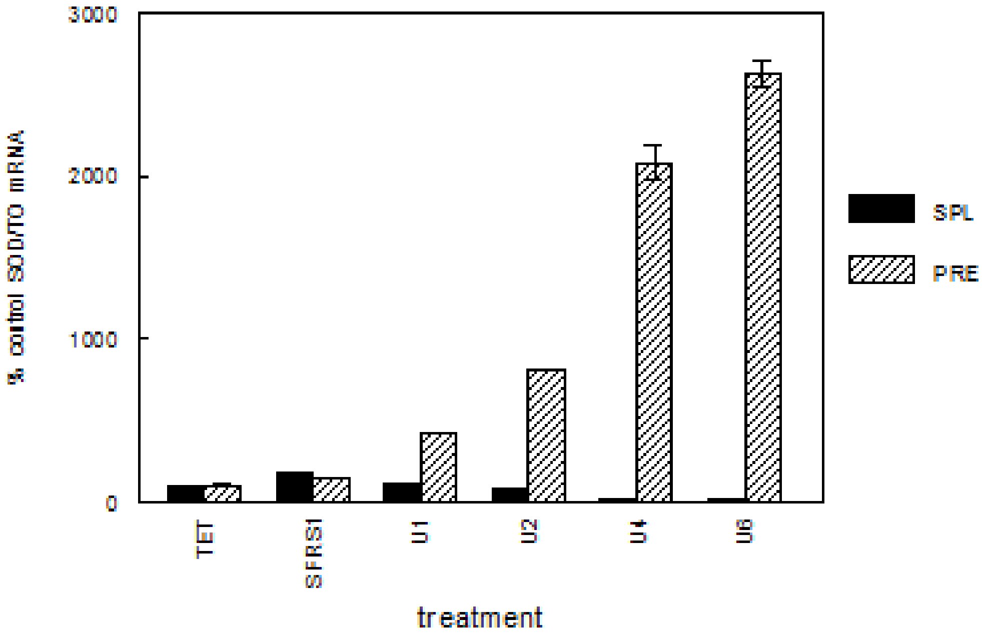

Supplement: Figure S7 — Effect of snRNA reduction on SOD1 minigene processing. A) Sequence of ASOs trageting snRNAs. ASOs are phosphorothioate at each position with MOE-substituted bases underlined. B) Northern analysis of U4/U6 snRNA reduction was carried out as previously described [25]. C) Effects of snRNA reduction on SOD1 minigene processing. SOD/TO cells were treated with ASOs targeting SRFS and snRNAs U1, U2, U4, and U6. After 24 hours minigene expression was induced by addition of TET to the media for 2 hours. Levels of minigene spliced and pre-mRNA were assessed by qRT/PCR using primer/probe set described previously [14]. Data is plotted as percent expression relative to mock treated control (TET) for spliced mRNA (solid bars) and pre-mRNA (striped bars). (PDF) [file pone.0110615.s007.pdf]
