# Supplementary material for: Targeting of Repeated Sequences Unique to a Gene Results in Significant Increases in Antisense Oligonucleotide Potency
Source: PLoS One. 2014 Oct 15;9(10):e110615. doi: 10.1371/journal.pone.0110615 (PMC4198294; doi:10.1371/journal.pone.0110615)
Supplement: Table S1 — Sequences of primers/probes used for qRT/PCR. For primers complementary to the minigene, vector sequence is in lower case. (PDF) [file pone.0110615.s008.pdf]

Table S1. Sequences of primers/probes used for qRT/PCR. For primers complementary to the minigene, vector sequence is in lower case.

Target RNA	Forward primer	Reverse primer	probe	location
SOD/GCGR minigene	taa tac gac tca cta tag gga ga	CTG CTT TTT CAT GGA CCA CCA	CAA AGA TGG TGT GGC CGA TG	Exon 4
SOD/GCGR minigene	TGG TGG TCC ATG AAA AAG CAG	ctg tgc tgg ata tct gca gaa ttc TTT AG	CGC TGG AAG TCG TTT GGC TTG TGG	Exon 5
GCGR	GACACCCCCGCCAATACC	CCGCATCTCTTGAACACGAA	TTGGCACCACAAAGT	Exon 4/5 junction
STAT3	GAGGCCCGCCCAACA	TTCTGCTAATGACGTTATCCAGTTTT	CTGCCTAGATCGGC	Exon 8/9 junction
STAT3	ACATGCCACTTTGGTGTTTCATAA	TCTTCGTAGATTGTGCTGATAGAGAAC	CAGTATAGCCGCTTCCTGCAAGAGTCGAA	Exon 3
TAU	AAGATTGGGTCCCTGGACAAT	AGCTTGTGGGTTTCAATCTTTTTATT	CACCCACGTCCCTGGCGGA	Exon 13/14 junction
OGFR	GCGCTTCCAGAACCTGAACT	CGACTTGAGGATGCGTGTGA	CGCAGCCACAACAACCTCCGC	Exon 6/7 junction
BOK	GCTGGTTGCTTAATCCGTTTCT	CCCCACCGCACAAGGA	AGGAAGAGTATGACACCCACTTGTGATGGG	Exon 5
