# Supplementary material for: Targeting of Repeated Sequences Unique to a Gene Results in Significant Increases in Antisense Oligonucleotide Potency
Source: PLoS One. 2014 Oct 15;9(10):e110615. doi: 10.1371/journal.pone.0110615 (PMC4198294; doi:10.1371/journal.pone.0110615)
Supplement: Table S2 — Sequences of primers used for insertion of GCGR site at non-tandem positions in the SOD1 minigene. (PDF) [file pone.0110615.s009.pdf]

Table S2. Sequences of primers used for insertion of *GCGR* site at non-tandem positions in the SOD1 minigene.

Primers for *GCGR* insertion	sense	antisense
Position 19	GCA TGT TGG AGA CTT GGT GGG CAC CTC GGG AAC CGG TGC AAT GTG ACT GCT GAC	GTC AGC AGT CAC ATT GCA CCG GTT CCC GAG GTG CCC ACC AAG TCT CCA ACA TGC
Position 334	GGG TAT TGT TGG GAG GAG TGG GCA CCT CGG GAA CCG GTG TAG TGA TTA CTT GAC AG	CTG TCA AGT AAT CAC TAC ACC GGT TCC CGA GGT GCC CAC TCC TCC CAA CAA TAC CC
Position 472	AAG ACA GGA AAC GCT GGT GGG CAC CTC GGG AAC CGG TAA GTC GTT TGG CTT GTG	CAC AAG CCA AAC GAC TTA CCG GTT CCC GAG GTG CCC ACC AGC GTT TCC TGT CTT
Position 523	GAT GTA GTC TGA GGC CCC TTA TGG GCA CCT CGG GAA CCG GTA CTC ATC TGT TAT CCT G	CAG GAT AAC AGA TGA GTA CCG GTT CCC GAG GTG CCC ATA AGG GGC CTC AGA CTA CAT C
