# Supplementary material for: Targeting of Repeated Sequences Unique to a Gene Results in Significant Increases in Antisense Oligonucleotide Potency
Source: PLoS One. 2014 Oct 15;9(10):e110615. doi: 10.1371/journal.pone.0110615 (PMC4198294; doi:10.1371/journal.pone.0110615)
Supplement: Table S3 — Sequences of ASOs complementary to GCGR. All ASOs are phosphorothioate at each position with MOE-substituted bases underlined. The number of sites is equal to the number of times the ASO is perfectly matched to the target sequence. Tm is calculated for RNA/DNA heteroduplexes [42]. (PDF) [file pone.0110615.s010.pdf]

Table S3. Sequences of ASOs complementary to *GCGR*. All ASOs are phosphorothioate at each position with MOE-substituted bases underlined.

ISIS #	*GCGR* ASO Sequence	length	# sites	Tm
398457	GGGTTCCCGAGGTGCCCAAT	20	8	80.52
436158	CAGGCTGTTGACAAAGCCCT	20	1	71.53
436159	GAGCTGGGCCCCCAGGATGG	20	1	84.49
436160	CTTTGCAGGGCTGGCACCCC	20	1	84.31
436161	CCACCTGAGCTCCACTGCCC	20	1	83.93
436162	CCACACCAGCCACCACCAGG	20	1	79.61
436163	CCCTTCCTCCCTCCCACAGC	20	1	88.67
436164	GGTTCCCGAGGTGCCCAATG	20	7	77.95
436165	CTTCTCAGGCCTTGGAGTCA	20	1	78.14
436166	AGAAACTCCCAGCTGTGGAG	20	1	69.07
436167	TCTCACCCAGCCCTCTCCCC	20	1	90.15
436168	GAAGAAGGGAGGCTCCTCTG	20	1	70.52
436169	CACTGGGTCTCTGATAGTGA	20	1	70.25
436170	CCCCCAGGCCTTTCTTTGAA	20	1	77.57
436171	CTGGCTCTGCCCCAACTCTG	20	1	79.38
436172	CCAGGAGCTCCCTGGGCAAA	20	1	77.36
436173	CTGATCAAACAGCCCCATCT	20	1	69.01
436174	GCCCTTGGACCCTGGGCTCA	20	1	86.56
436175	AGCTGGAAGGCAGTGCCAGG	20	1	77.42
436176	GAATAAGAGGTGTGGATGGA	20	1	60.46
436177	CAGCATCTGAGCTGGGAGTT	20	1	74.34
436178	GGCTCGGTTGGGCTTCCTGG	20	1	83.91
436179	GGCTGGCTTGCAGCTGTGCC	20	1	86.99
436180	TTGCCAGGCTGACCTGAGCC	20	1	80.66
436181	ATTCTTAGCCCCTGGGAAAG	20	1	69.01
436182	CCCTCAGTTGACAGCCCCCG	20	1	82.54
436183	GGGACCAGCCTCCCCAGATG	20	1	82.17
436185	GGCTGGCAGCTCTGAGGGTC	20	1	85.40
449874	GTTTTTGTGAAACCAGA	17	1	54.77
449875	TGTTTTTGTGAAACCAG	17	1	53.23
449881	TCCCGAGGTGCCCAATG	17	7	71.74
449882	TTCCCGAGGTGCCCAAT	17	8	72.29
449883	GTTCCCGAGGTGCCCAA	17	8	75.92
449884	GGTTCCCGAGGTGCCCA	17	8	81.53
449885	GGGTTCCCGAGGTGCCC	17	8	83.28
449887	GTCCAGGTGACCAAGCT	17	1	72.01
449888	CAGTCCAGGTGACCAAG	17	1	66.43
449889	ACAGTCCAGGTGACCAA	17	1	66.81
