# Supplementary material for: Targeting of Repeated Sequences Unique to a Gene Results in Significant Increases in Antisense Oligonucleotide Potency
Source: PLoS One. 2014 Oct 15;9(10):e110615. doi: 10.1371/journal.pone.0110615 (PMC4198294; doi:10.1371/journal.pone.0110615)
Supplement: Table S4 — Sequences of ASOs complementary to STAT3. All ASOs are phosphorothioate at each position with cEt-substituted bases underlined. The number of sites is equal to the number of times the ASO is perfectly matched to the target sequence. (PDF) [file pone.0110615.s011.pdf]

Table S4. Sequences of ASOs complementary to *STAT3*. All ASOs are phosphorothioate at each position with cEt-substituted bases underlined.

IsisNo	*STAT3* ASO Sequence	Length	# sites	Tm
691621	AATATTCACTTGCCTC	16	1	56.71
691623	CTAATATTCACTTGCC	16	1	54.43
691625	GAGTCTTTCAACTCAA	16	1	53.90
691627	AAATGAGTCTTTCAAC	16	1	46.08
691631	TTAAGATCTAAACAGA	16	1	41.54
691633	GCCTTAAGATCTAAAC	16	1	49.91
691635	CCGCCCGCCTTAAGAT	16	1	69.13
691637	GCCCCGCCCGCCTTAA	16	1	79.16
691641	CCTCCCTTGCGCCCCG	16	1	81.52
691645	TTTGAGGACCCGTACC	16	1	65.33
691647	CCTTTGAGGACCCGTA	16	1	66.91
691649	GGCTCCTTTGAGGACC	16	1	71.53
691651	TAATGACCAGGCTCCT	16	1	63.84
691653	GTCCTTAATGACCAGG	16	1	60.69
691655	CTGTCCTTAATGACCA	16	1	59.60
691657	TACTCCTGTCCTTAAT	16	1	59.92
691659	GGGAATACTCCTGTCC	16	1	65.81
691662	TGAGGGAATACTCCTG	16	27	57.40
691664	CCTGAGGGAATACTCC	16	35	62.09
691666	GACCTGAGGGAATACT	16	36	58.10
691668	TGGACCTGAGGGAATA	16	26	58.12
691669	CTGGACCTGAGGGAAT	16	26	61.08
691671	TCCTGGACCTGAGGGA	16	26	70.00
691675	ATACTCCTGGACCTGA	16	26	64.16
691677	GAATACTCCTGGACCT	16	26	61.75
691679	GGGAATACTCCTGGAC	16	26	61.02
691681	GAGGGAATACTCCTGG	16	26	60.60
691682	TGACCTGAGGGAATAC	16	11	55.71
691686	TCCTTGACCTGAGGGA	16	9	67.29
691689	TACTCCTTGACCTGAG	16	8	61.72
691691	AATACTCCTTGACCTG	16	8	57.47
691693	GGAATACTCCTTGACC	16	8	59.85
691695	AGGGAATACTCCTTGA	16	8	57.88
691697	TGAGGGAATACTCCTT	16	8	57.88
