# Supplementary material for: Targeting of Repeated Sequences Unique to a Gene Results in Significant Increases in Antisense Oligonucleotide Potency
Source: PLoS One. 2014 Oct 15;9(10):e110615. doi: 10.1371/journal.pone.0110615 (PMC4198294; doi:10.1371/journal.pone.0110615)
Supplement: Table S5 — Sequences of ASOs complementary to MAPT. All ASOs are phosphorothioate at each position with MOE-substituted bases underlined. The number of sites is equal to the number of times the ASO is perfectly matched to the target sequence. (PDF) [file pone.0110615.s012.pdf]

Table S5. Sequences of ASOs complementary to *MAPT*. All ASOs are phosphorothioate at each position with MOE-substituted bases underlined.

ISIS #	*MAPT* ASO Sequence	length	# sites	Tm
620887	GTTTTCAAACACACCTTC	18	2	58.59
620888	ACACCTTCATTTACTGTC	18	2	62.84
620889	TTTTCAAACACACCTTCA	18	2	56.74
620890	GGTTTTCAAACACACCTT	18	2	59.88
620891	TGGTTTTCAAACACACCT	18	2	59.44
621345	CAACTGCTCTTCCCTGGG	18	1	72.75
621346	TCTGGGTGCAGTTTATGC	18	1	70.34
621347	AGAATTTATGCTGGAAAT	18	1	49.39
621348	GGCCAGCTCTCCAAATCC	18	1	74.24
621349	CCCTAGACTCTGGGCAAC	18	1	70.43
621350	CCAGGAGATGAAGTAGCA	18	1	62.80
621353	CTGCCTGGCCAATTAAAT	18	1	61.49
621354	CAAACTAATATTAACACT	18	1	43.29
621355	TACTCAATTCCAGGCAAG	18	1	60.03
621356	AATGGAAGTTCTAGTACG	18	1	53.83
621357	CAATCACAGTTCTTTTTC	18	1	57.31
621358	GTCTTAAACATAAACATA	18	1	44.59
623672	GTGGATGTCTTAAACATA	18	1	53.94
625423	ACCTTCATTTACTGTCAG	18	1	62.45
625424	CACCTTCATTTACTGTCA	18	1	63.59
625425	CACACCTTCATTTACTGT	18	2	62.59
625426	ACACACCTTCATTTACTG	18	2	59.80
625427	AACACACCTTCATTTACT	18	2	57.72
625428	AAACACACCTTCATTTAC	18	2	53.66
625429	CAAACACACCTTCATTTA	18	2	54.47
625430	TCAAACACACCTTCATTT	18	2	56.39
625431	TTCAAACACACCTTCATT	18	2	56.39
625432	TTTCAAACACACCTTCAT	18	2	56.39
625433	TTGGTTTTCAAACACACC	18	1	57.74
