# Supplementary material for: Targeting of Repeated Sequences Unique to a Gene Results in Significant Increases in Antisense Oligonucleotide Potency
Source: PLoS One. 2014 Oct 15;9(10):e110615. doi: 10.1371/journal.pone.0110615 (PMC4198294; doi:10.1371/journal.pone.0110615)
Supplement: Table S6 — Sequences of ASOs complementary to OGFR. All ASOs are phosphorothioate at each position with MOE-substituted bases underlined. (PDF) [file pone.0110615.s013.pdf]

Table S6. Sequences of ASOs complementary to *OGFR*. All ASOs are phosphorothioate at each position with MOE-substituted bases underlined.

| ISIS # | *OGFR* ASO Sequence | length | #sites | Tm |
|---|---|---|---|---|
| 207558 | GACCCTGTCATTCTGGACTG | 20 | 1 | 72.95 |
| 207561 | GTCTCCGATGGGCTCTCGGC | 20 | 6 | 82.56 |
| 207563 | GCCTCGGTCCTCCAGCCGGA | 20 | 1 | 87.78 |
| 207565 | CTCCGATGGGCTCTCGGCTG | 20 | 6 | 79.01 |
| 207572 | CCCGGACAAGCCGCTCCTGG | 20 | 1 | 79.14 |
| 207575 | GCACTCTGAGGGCCTGGCTC | 20 | 1 | 84.70 |
| 207590 | GGCCCCGTTCGACCTTCTGT | 20 | 1 | 82.70 |
| 696868 | GGCTCGTCCCCTGCAGGTCC | 20 | 5 | 90.73 |
| 696869 | CGGCTGGCTCGTCCCCTGCA | 20 | 5 | 86.74 |
| 696870 | CCGATGGGCTCTCGGCTGGC | 20 | 6 | 82.08 |
| 696871 | GGGTCTCCGATGGGCTCTCG | 20 | 6 | 80.74 |
| 696878 | CCCTGCAGGTCCTGCCGGGC | 20 | 3 | 89.81 |
| 696879 | CCCCTGCAGGTCCTGCCGGG | 20 | 3 | 88.71 |
| 696880 | CCCAGGTGGAGTCGCAGTCG | 20 | 1 | 79.86 |
| 696881 | ACTGGAACGAGCTGGGCCGC | 20 | 1 | 74.96 |
| 696882 | CAGCCGTTGGGCAGGAAGCG | 20 | 1 | 75.28 |
| 696884 | GCGCCTGGAAGTGCTCGAGG | 20 | 1 | 76.50 |
| 696885 | AGTGCACCAGCTGGCGGCGC | 20 | 1 | 83.74 |
| 696886 | TCCTGGAGCCCTCGAGCGGA | 20 | 1 | 81.18 |
| 696887 | TCCGACCCTGGGTGCTGGCC | 20 | 1 | 85.96 |
| 696888 | GGCCTATCTTCCCCGTGGCC | 20 | 1 | 86.90 |
| 696889 | GGCGGCAGGGCTGCACTGCC | 20 | 1 | 87.39 |
| 696890 | TCTGGGCACCACCACTGGCC | 20 | 1 | 82.88 |
| 696891 | AGTGGCCCCACAGGAGGCCA | 20 | 1 | 85.48 |
| 696892 | CAGCCAGGCCAGGAGGCTTC | 20 | 1 | 83.71 |
| 696893 | GTCCAGGGCACTCTGCCGCA | 20 | 1 | 85.44 |
