# Supplementary material for: Targeting of Repeated Sequences Unique to a Gene Results in Significant Increases in Antisense Oligonucleotide Potency
Source: PLoS One. 2014 Oct 15;9(10):e110615. doi: 10.1371/journal.pone.0110615 (PMC4198294; doi:10.1371/journal.pone.0110615)
Supplement: Table S7 — Sequences of ASOs complementary to BOK. All ASOs are phosphorothioate at each position with MOE-substituted bases underlined. (PDF) [file pone.0110615.s014.pdf]

Table S7. Sequences of ASOs complementary to *BOK*. All ASOs are phosphorothioate at each position with MOE-substituted bases underlined.

IsisNo	*BOK* ASO Sequence	Length	# sites	Tm
239733	GTGTTCCCGGGCCCCTTCAC	20	6	86.82
239736	CTCAGGTGAGAGCAGGTGTT	20	9	75.32
239742	GTTCAGGTGAGAGCAGGTGT	20	2	77.01
239744	GTGAGAGCAGGTGTTCCCGG	20	5	78.98
239751	TTCCGGGCCCCTTCACCTGG	20	6	84.22
239756	GCTCAGGTGAGAGCAGGTGT	20	9	79.76
703841	TCAGGTGAGAGCAGGTGTTC	20	11	75.05
703842	GGTGTTCCCGGGCCCCTTCA	20	6	88.67
703843	AGGTGTTCCCGGGCCCCTTC	20	6	88.15
703844	AGCAGGTGTTCCCGGGCCCC	20	5	89.61
703845	AGAGCAGGTGTTCCCGGGCC	20	5	84.77
703846	CAGGTGAGAGCAGGTGTTCC	20	9	77.10
703847	GTGTTCCGGGCCCCTTCACC	20	8	86.82
703848	GGTGTTCCGGGCCCCTTCAC	20	6	86.07
703849	CAGGTGTTCCGGGCCCCTTC	20	4	85.83
703850	GCAGGTGTTCCGGGCCCCTT	20	4	88.28
703851	AGGTGAGAGCAGGTGTTCCG	20	4	75.67
703852	CCCAGCTGGCGAGAGGCTGG	20	1	81.38
703853	TTCAGGATCTGCTCGCCGCC	20	1	79.76
703854	GCTCATCGCCCAGGCGCAGC	20	1	85.39
703855	GTGGCCAGCCACGGCCAGGA	20	1	85.44
703856	GCGGCCACCGCATACAGGGA	20	1	78.38
703857	GGACCATGGCAGGCTGGGCC	20	1	84.61
703858	CCGCAGCCAGGTTGCCAGGG	20	1	84.33
703859	GCCACCAGCCAGTGGGAGCG	20	1	83.36
703860	GCCAGGCTGCGGCCACTGCC	20	1	88.83
703861	ACCCCTTCCGGAGGGCCTGG	20	1	85.15
703862	GGAGGGTCGCAGGCTGCCCC	20	1	89.19
703863	GGCCTGGGAGTTGACCCTGA	20	1	80.55
703864	CAGCCAGGCCAGGGTCACCT	20	1	85.81
703865	TGGCCTGGAGCCCGCCGGCC	20	1	89.46
703866	ATTCCTTCTCGGCCGTAGGT	20	1	78.30
703867	GACTGCCCCATGTCCAGCCT	20	1	84.89
703868	GCACGAAGCCTGGCCGGGAG	20	1	77.28
